# CDK2 regulates collapsed replication fork repair in CCNE1-amplified ovarian cancer cells via homologous recombination

**DOI:** 10.1093/narcan/zcad039

**Published:** 2023-07-27

**Authors:** Victoria E Brown, Sydney L Moore, Maxine Chen, Nealia House, Philip Ramsden, Hsin-Jung Wu, Scott Ribich, Alexandra R Grassian, Yoon Jong Choi

**Affiliations:** Blueprint Medicines, Cambridge, MA 02139, USA; Department of Biology, Tufts University, Medford, MA 02155, USA; Blueprint Medicines, Cambridge, MA 02139, USA; Department of Biology, Tufts University, Medford, MA 02155, USA; Blueprint Medicines, Cambridge, MA 02139, USA; Blueprint Medicines, Cambridge, MA 02139, USA; Blueprint Medicines, Cambridge, MA 02139, USA; Blueprint Medicines, Cambridge, MA 02139, USA; Blueprint Medicines, Cambridge, MA 02139, USA; Blueprint Medicines, Cambridge, MA 02139, USA; Blueprint Medicines, Cambridge, MA 02139, USA

## Abstract

*CCNE1* amplification is a common alteration in high-grade serous ovarian cancer and occurs in 15–20% of these tumors. These amplifications are mutually exclusive with homologous recombination deficiency, and, as they have intact homologous recombination, are intrinsically resistant to poly (ADP-ribose) polymerase inhibitors or chemotherapy agents. Understanding the molecular mechanisms that lead to this mutual exclusivity may reveal therapeutic vulnerabilities that could be leveraged in the clinic in this still underserved patient population. Here, we demonstrate that *CCNE1*-amplified high-grade serous ovarian cancer cells rely on homologous recombination to repair collapsed replication forks. Cyclin-dependent kinase 2, the canonical partner of cyclin E1, uniquely regulates homologous recombination in this genetic context, and as such cyclin-dependent kinase 2 inhibition synergizes with DNA damaging agents *in vitro* and *in vivo*. We demonstrate that combining a selective cyclin-dependent kinase 2 inhibitor with a DNA damaging agent could be a powerful tool in the clinic for high-grade serous ovarian cancer.

## Graphical Abstract

**Graphical Abstract:**
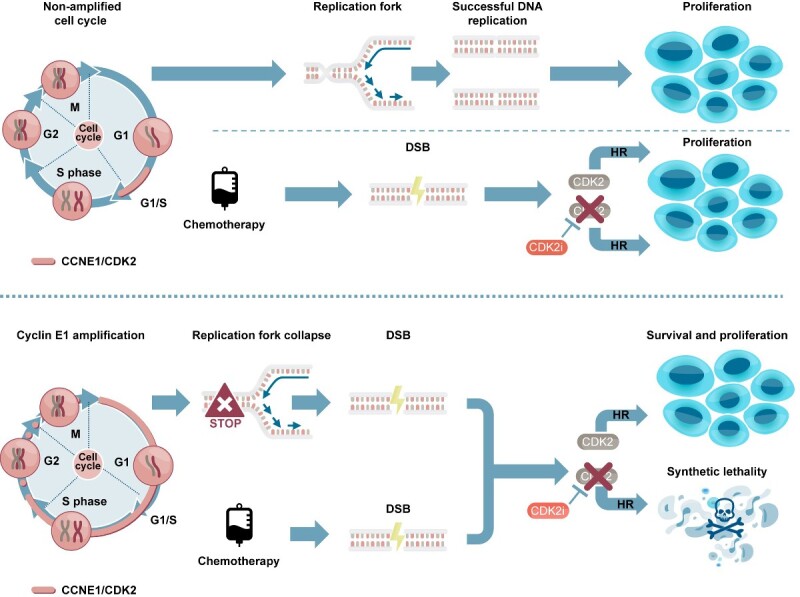

## INTRODUCTION

Ovarian cancer is the leading cause of death from gynecologic cancer, and the fifth leading cause of cancer-related deaths in women in the United States; in 2020, approximately 200 000 women worldwide died from ovarian cancer (1). This disease generally has a poor prognosis, with a 5-year survival rate of only one-third of patients (1). Patients with homologous recombination deficient (HRD) ovarian cancer, such as those with mutations in *BRCA1/2*, have showed clinical benefit from poly (ADP-ribose) polymerase (PARP) inhibitors, due to the synthetic lethal relationship between alternative non-homologous end joining (NHEJ), a pathway controlled in part by PARP, and homologous recombination (HR) (2,3). It has been previously reported that *CCNE1*-amplification, which occurs in 15–20% of high-grade serous ovarian cancer (HGSOC) patient tumors, generally does not co-occur with loss of function mutations of HR genes (4,5). It is believed that because *CCNE1*-amplified tumors have intact HR, they are resistant to PARP inhibitors and platinum agents, which is the standard of care for HGSOC (6). HGSOC patients with *CCNE1-*amplified tumors, therefore, have limited therapeutic options and represent a high unmet medical need.

Cyclin-dependent kinase 2 (CDK2) is the canonical partner of cyclin E1, and this complex controls entry into the S-phase (7) via phosphorylation of the retinoblastoma protein 1 (RB1) to release E2F and allow for transcription of genes that will drive the cell cycle (8). Aberrant activation of the RB1-E2F pathway, such as through overexpression of *CCNE1*, increases double-stranded break (DSB) formation, leading to genomic instability (9,10). Specifically, *CCNE1* overexpression induces premature S-phase entry (11), increasing replication fork stalling and collapse via insufficient nucleotide pools (12), or replication-transcription collisions stemming from inappropriate origin firing (13). Although some studies have demonstrated a synthetic lethality between *CCNE1* amplification and HRD, such as through genetic suppression of *BRCA1/2* (14), other large-scale screens have not supported this relationship (15,16). The discrepancy in these results may stem from the experimental conditions (e.g. clonogenic assays versus logarithmic growth). More investigation is required to understand the molecular mechanism(s) underlying the mutual exclusivity of these two genomic events.

In addition to their role in cell cycle progression, the cyclin-dependent kinases (CDKs) are known regulators of HR in eukaryotic cells. Cyclin-dependent kinase 1 (CDK1) and CDK2 have been implicated in HR in human cells via substrate phosphorylation during each step of recombination, but with conflicting evidence to support whether cells are dependent on CDK1, CDK2 or both for this pathway (17–24). These studies are in part confounded by the use of non-selective CDK inhibitors, making it difficult to ascribe specific functions to particular CDK family members. Moreover, the role of CDKs in DNA repair has not been well studied in a context where the core cell cycle machinery is dysregulated, as is the case in *CCNE1*-amplified cells.

Here, we provide evidence for a mechanism that could explain the mutual exclusivity of *CCNE1* amplification and HRD in HGSOC. We demonstrate that *CCNE1*-amplified HGSOC cells rely on HR to resume replication following induced fork collapse. We detected high levels of CDK2 and cyclin E1 at the site of stalled replication forks only in *CCNE1*-amplified cells, which places CDK2-cyclin E1 directly at barriers to replication. Lastly, we provide evidence that CDK2 is required for both signaling after replication fork stalling and HR in *CCNE1*-amplfied cells, but is dispensable in *CCNE1* non-amplified cells. Inhibition of CDK2 at low doses sensitizes *CCNE1*-amplified cells to DNA-damaging agents both *in vitro* and *in vivo*. With the advancement of CDK2 inhibitors into the clinic, these studies suggest that the combination of CDK2 inhibition with DNA-damaging chemotherapy or PARP inhibitors may provide clinical benefit in HGSOC patients with *CCNE1*-amplified tumors.

## MATERIALS AND METHODS

### Cell lines and culture

OVCAR-3 cells were maintained in Roswell Park Memorial Institute (RPMI) 1640 media supplemented with 20% fetal bovine serum. hTERT-RPE1 cells were maintained in Dulbecco's Modified Eagle Medium: F12 (DMEM: F12) supplemented with 10% fetal bovine serum and 0.01 mg/ml hygromycin B. A549 cells were maintained in F-12K Medium supplemented with 10% fetal bovine serum. COV644 and COV318 cells were maintained in DMEM supplemented with 10% fetal bovine serum. FUOV1 cells were maintained in Ham's F12 + DMEM at 1:1, and supplemented with 10% fetal bovine serum. MCF-7 cells were maintained in Eagle's Minimum Essential Medium (EMEM), and supplemented with 0.01 mg/ml human recombinant insulin and 10% fetal bovine serum. OVCAR-3, hTERT-RPE1, A549, and MCF-7 cells were purchased from the American Type Culture Collection. COV644 and COV318 were purchased from Sigma Aldrich. FUOV1 cells were purchased from DSMZ. Cell line identity was confirmed with short tandem repeat (STR) testing (Charles River). All cells were maintained in a humidified incubator at 37°C with 5% CO_2_.

### Proliferation assay

OVCAR-3, COV644, or MCF-7 cells were seeded at 1000 cells/well in 384-well black, clear bottom plates and allowed to adhere overnight at 37°C/5% CO_2_. The following day, cells were treated with compound in a 10-point dose response, 1:4 dilutions. After 5 days of incubation, CyQuant Direct Proliferation Assay was performed according to the manufacturer's instructions. Data were processed with GraphPad Prism using a four-parameter fit to calculate IC_50_.

### AlphaLISA assays

For the RB1 phosphorylation (T821/826) AlphaLISA, OVCAR-3 cells were seeded in serum-free DMEM in a 96-well plate (25 000 cells/well) the day before the assay. After overnight incubation, media were replaced with DMEM containing 10% FBS, and cells were treated with a dose response of compound (1:4 dilutions) for 18 h. pRb AlphaLISA was performed according to the manufacturer's instructions. Plates were read on EnVision plate reader (Perkin Elmer). Data were processed with GraphPad Prism using a four-parameter fit to calculate IC_50_.

For the Lamin phosphorylation (S22) AlphaLISA, OVCAR-3 or COV644 cells were seeded in DMEM containing 10% FBS in 384-well plates (3000 cells/well for OVCAR-3; 12 500 cells/well for COV644). After overnight incubation, cells were treated with a dose response of compound (1:4 dilutions) for 2 h. Media was aspirated from the plate, and cells were lysed in 10 μl of 1× AlphaLISA lysis buffer (Perkin Elmer Cat# AL003C) and placed on a plate shaker for 15 min. Anti-total lamin (SC-7292) was diluted to 0.2 nM, and anti-phospho Lamin (CST# 13448) was diluted to 0.3 nM in 1× immunoassay buffer (Perkin Elmer Cat# AL00F). 10 μl of each antibody was added to the plate and incubated for 1 h at RT. Anti-rabbit acceptor beads (Perkin Elmer Cat# AL104R) were prepared at 5× and added to the plate (10 μl/well) and incubated for 1 h at RT. Anti-mouse donor beads (Perkin Elmer Cat# AS104R) were prepared at 5× and added to the plate (10 μl/well) and incubated overnight in the dark. Plates were read on EnVision plate reader (Perkin Elmer). Data were processed with GraphPad Prism using a four-parameter fit to calculate IC_50_.

### Lentiviral transduction/stable cell line generation

Lentivirus encoding inducible non-targeting control shRNA, CDK2 shRNA or CDK1 shRNA in the SMARTvector inducible lentiviral shRNA vector under the EF1 alpha promoter with puromycin resistance were purchased from Horizon Discovery. Stable OVCAR-3, FUOV1, COV318, hTERT-RPE1, A549 and COV644 cells were generated by exposing cells to lentivirus at a multiplicity of infection of 1:1 overnight in the presence of 5 μg/ml polybrene. After overnight incubation, media was replaced and cells were allowed to recover for 48 h. Cells were passaged into selection media (Puromycin – OVCAR-3: 2 μg/ml, FUOV1: 0.5 μg/ml, COV318: 5 μg/ml, hTERT-RPE1: 10 μg/ml, A549 and COV644: 1 μg/ml) for 2 weeks before experiments were conducted.

### Western blot

Cells were lysed in PhosphoSafe Lysis Buffer (EDM millipore, Cat#71296) and protein was quantified using the Pierce BCA Gold Kit (Thermo Fisher Scientific Cat#A53227). Lysate and lysis buffer were combined with 4× Loading Dye (Bio-Rad, Cat#1610747) and 2ME (Bio-Rad, Cat#1610710). Samples were loaded into Bio-Rad 4–20% Tris-glycine, 18-well SDS-PAGE (Cat#5671094). Transfer was performed using Bio-Rad dry transfer system (Trans-Blot Turbo) (Nitrocellulose membrane, 0.2 μM). Blocking was performed using LI-COR Odyssey Blocking Buffer (Cat#927–70001). Primary antibodies (CDK2 1:500, p16 1:1000, CCNE1 1:500, pRbT821 1:500, Actin R 1:2000, p21 1:1000, CCNA2 1:500, pRb S807/811 1:1000, pRb S780 1:500, CtIP 1:1000, Actin M 1:5000, pH2A.X Ser139 1:1000, pCDK2 T160 1:1000, pRPA S4/8 1:1000, CDK1 1:1000, pCDK1 Y15 1:1000, pChk1 S317 1:500, Total Chk1 1:1000, Tubulin 1:10 000) were combined with Odyssey Blocking Buffer and 0.1% Tween-20, and incubated at 4°C, rotating. Secondary antibodies were combined with Odyssey Blocking Buffer and 0.1% Tween-20 and incubated at room temperature, rotating. All secondary antibodies were diluted to 1:10 000 (LI-COR Cat#926–33212, Cat#926-33213, Cat#926-68070, Cat#926–68071, Cat#926–68076). Signals were detected using LI-COR Odyssey CLx Imaging Studio.

### CRISPR/cas9 recombination assay

pBluescript SK II + and LentiCRISPR v2 plasmids were purchased from Genscript. LentiCRISPR v2 plasmids expressing single guide (sgRNA) against ACTB or scrambled sgRNA were constructed according to the depositor's instructions. To construct HR donor vectors against ACTB, sequences from −1554 to +1527 relative to the stop codon of ACTB plus 3× FLAG directly 5’ from the stop codon were synthesized and cloned into pBluescript SK II using restriction digest and ligation. Compounds were added to cells on the day of plasmid transfection. sgRNA/Cas9 and the donor plasmid were prepared in a 1:2 molar ratio; 1 μg of total DNA/well was transfected onto cells using Lipofectamine 2000 (Thermo Fisher Scientific Cat#11668019). Cells were harvested for Western Blot analysis 72 h later.

### *In situ* proximity ligation assay for EdU (double stranded DNA)–protein interaction

Cells were seeded onto microscope chamber slides the day before the experiment in DMEM supplemented with 10% FBS. On the day of the experiment, cells were incubated with 100 μM EdU for 10 min and treated with hydroxyurea (2 mM) for 2 h. After treatment, cells were pre-extracted in CSK-100 buffer (100 mM NaCl, 300 mM sucrose, 3 mM MgCl_2,_10 mM Pipes pH 6.8, 1 mM EGTA, 0.2% Triton X-100, 1 × antiproteases) for 5 min on ice under gentle agitation and fixed with 4% PFA/PBS for 20 min at room temperature (RT). Cells were permeabilized in 0.2% Triton X-100/PBS for 10 min at RT. Cells were incubated for 30 min at RT with Click-iT reaction cocktail (Thermo Fisher, Cat#C10269) to attach a biotin azide (ThermoFisher, Cat# B10184) to EdU. Next, cells were incubated with PLA blocking buffer (Sigma Aldrich) for 1 h at 37 °C in a humidified chamber before addition of primary antibodies: rabbit anti-CCNE1 (Sigma-Aldrich, 1:250); mouse anti-CDK2 (Santa Cruz,1:50); mouse anti-CCNA2 (Santa Cruz, 1:800); rabbit anti-biotin (CST,1:200), and mouse anti-biotin (Sigma, 1:100). The negative control used only one primary antibody. Slides were incubated with secondary antibodies conjugated with PLA probes MINUS (anti-rabbit) and PLUS (anti-mouse) (Millipore Sigma). The incubation with all antibodies was done in a humidified chamber for 1 h at 37°C. PLA probes MINUS and PLUS were ligated using two connecting oligonucleotides to form a template for rolling‐cycle amplification. Following amplification, the products were hybridized with red fluorescence‐labeled oligonucleotide. Samples were mounted in Duolink *In Situ* mounting medium with DAPI (blue) (Sigma-Aldrich, Cat# DUO82040). Images were acquired using the Nikon Ti inverted, spinning disk confocal microscope ‘Spinster’ in the MicRoN (Microscopy Resources on the North Quad) core at Harvard Medical School. PLA spots per nuclei were quantified in each plane of the Z-stack using arivis Vision4D analysis software on the MicRoN Big Dipper Image Analysis work station. Images used in manuscript represent maximum projects of a Z-stack from ImageJ FIJI.

### Enzyme assays

All CDK/cyclin assays were performed with 1 mM ATP. Untreated enzyme (0.65 nM for CDK2/cyclin E, 5 nM for CDK2/cyclin A2, 0.60 nM for CDK1/cyclin B, 1 nM for CDK4/cyclin D1, 1 nM for CDK6/cyclin D3, 15.0 nM for CDK7/cyclin H1/MNAT1, 3.0 nM for CDK9/cyclin T1) was incubated for 30 min in the presence or absence of compound. For CDK2/cyclin E and CDK1/cyclin B, the buffer used was 20 mM HEPES, pH 7.5, 0.0015% Brij 3510 mM MgCl_2_, 0.01% BSA, and 2.0 mM DTT. For CDK2/cyclin A2, the buffer used was 50 mM HEPES, pH 7.5, 10 mM MgCl_2_, 0.0015% Brij 35, and 2 mM DTT. For CDK7/cyclin H1/MNAT1 and CDK9/cyclin T1, the buffer used was 20 mM HEPES, pH 7.5, 0.01% Triton X-100, 10 mM MgCl_2_, 0.01% BSA and 2 mM DTT. For CDK4/cyclin D1 and CDK6/cyclin D3, the buffer used was 20 mM HEPES, pH 7.5, 0.0015% Brij 35, 10 mM MgCl_2_, 0.01% BSA and 2.0 mM DTT. After enzyme-inhibitor preincubation, the kinase reaction was initiated by the addition of fluorescent peptide substrate (for CDK2/cyclin E and CDK1/cyclin B, 3 μM ProfilerPro Kinase Substrate 18, 5-FAM-QSPKKG-CONH2). For CDK7/cyclin H1/MNAT1 and CDK9/cyclin T1, 5 μM CTD3. For CDK4/cyclin D1 and CDK6/cyclin D3, 1 μM ProfilerPro Kinase Substrate 34, 5-FAM-RRRFRPASPLRGPPK-COOH, and 1.0 mM ATP at 28°C for 30–60 min or until 10–20% total substrate peptide is phosphorylated in the absence of inhibitor. The kinase reactions were stopped by the addition of stop Buffer (75 mM HEPES, pH 7.5, 0.01125% Brij 35, 37.5 mM EDTA and 0.15% of Coating Reagent 3 [Perkin Elmer]). The plates were read on a Caliper EZReader. Data were normalized to 0% inhibition (DMSO) and 100% inhibition (10 μM staurosporine) controls, and the IC_50_ calculated using XL fit.

### NanoBRET target engagement assays

HEK293 cells were prepared to a final concentration of 100 000 cells/ml in 1% fetal bovine serum (FBS) Opti-MEM. Transfection mixtures were prepared with 1 μg of plasmid encoding NanoLuc-fused CDK and 9 μg of plasmid encoding cyclin, diluted in 1 ml Opti-MEM. FuGENE HD (Promega) was added to transfection mixture at a ratio of 30 μl/1 ml. After liposome formation, the lipid:DNA complex was added at a 1:20 ratio to HEK293 cells in suspension. Cells were plated in 384-well tissue culture plates and incubated overnight (20–30 h) at 37°C/5% CO_2_. Compounds were prepared from DMSO stock solutions and added to cell plates with a final top concentration of 10 000 nM. NanoBRET tracer solution was prepared from 100X tracer solution. For CDK1/cyclin B1, CDK9/cyclin T1, and CDK2/cyclin E1 assays, tracer K10 was used at a final concentration of 0.5 μM, 0.250 μM, and 0.125 μM, respectively. For CDK4/cyclin D1, and CDK6/cyclin D3, tracer K7 was used at a final concentration of 0.0625 μM. For CDK7/cyclin H1, tracer K7 was used at a final concentration of 0.5 μM. The plate was incubated at 37°C/5% CO_2_ for 2 h. NanoBRET substrate solution was prepared 15 min prior to the assay endpoint using serum-free OptiMEM. After the incubation was complete, NanoBRET substrate solution was added to the treated cell plate. The plate was then shaken and read using the Perkin Elmer Envision plate reader. The donor emission was measured at 450 nm, followed by acceptor emission at 610 nm. BRET ratio was generated by dividing the acceptor emission signal by the donor emission signal per sample, and converted to milliBRET by multiplying each raw value by 1000. Data were normalized to no drug (100% signal) and no tracer (0% signal), and fit using GraphPad Prism v8.0.2.

### Cell cycle profiling

Reagents for EdU incorporation were included in the Click-iT EdU Alexa Fluor 488 Flow Cytometry Assay Kit (Thermo Fisher Scientific Cat#C10420). Cells were treated with compound for 22 h prior to EdU pulsing. EdU was added to cells in culture for a final concentration of 10 μM, and incubated at 37°C in 5% CO_2_ for 2 h prior to collection and pelleting. Cells were incubated in the dark for 15 min with 100 μl of Click-iT fixative, washed with 1% BSA PBS, and incubated for 15 min with 100 μl 1x Click-iT saponin-based permeabilization agent, prepared by 1:10 dilution of stock solution in PBS supplemented with 1% BSA. The Click-iT reaction cocktail was prepared following manufacturer's instructions and added to cells for 30 min at room temperature in the dark. Cells were washed with 1% BSA. FxCycle Violet (Thermo Fisher Scientific Cat#F10347) was prepared in 1× saponin-based permeabilization reagent at a 1:2000 dilution, and added to cells for 30 min at room temperature in the dark. FACS analysis was performed using LSR Fortessa (BD Biosciences) and analyzed using FlowJo.

### Synergy analysis

OVCAR-3 or COV644 cells were seeded in 96-well plates in complete media. The next day, BLU1851 was added in a 10-point dose response. After 24 h of incubation, etoposide was added in a five-point dose response. The following day, compound containing media was aspirated, cells washed, and complete media added back. CyQuant was used 3 days later to determine cellular proliferation. HSA scores were determined using Synergy Finger (https://synergyfinder.org/).

### In vivo tumor growth inhibition, pharmacokinetics, and pharmacodynamics

Female NOD-SCID mice were purchased from Shanghai Lingchang BioTech Co., Ltd at 6–8 weeks old. Mice were housed in solid bottom polycarbonate ventilated cages at a temperature of 20–26°C in 40–70% humidity. Mice were kept on a 12-h light,

Twelve-hour dark cycle with regularly changed 100% filtered air. Municipal water and rodent diet were provided ad libitum. The general health of the animals was monitored through gross observation, regular body weight measurement, and monitoring of food and water intake. All procedures related to animal handling, care, and treatment were performed according to the protocol approved by the Institutional Animal Care and Use Committee of Shanghai Chempartner following the guidance of the Association for Assessment and Accreditation of Laboratory Animal Care (AAALAC).

OVCAR-3 cells were passaged through mice once, and a cell line was rederived from the tumor, named ‘T2A.’ OVCAR-3 T2A cells were cultured in RPMI 1640 media supplemented with 20% FBS and 0.01 mg/ml insulin. To establish the OVCAR-3 xenograft model, 6 × 10^6^ cells per mouse were implanted subcutaneously into the right hind flank of NOD-SCID female mice. Cells were suspended in 50% Matrigel and 50% RPMI 1640 media serum free media prior to implantation.

In OVCAR-3 tumor growth inhibition studies, animals were randomized and treatment began when tumors reached ∼200 mm^3^ in size (*n* = at least eight/group). BLU2256 was prepared in 20% (v/v) PEG 400, 80% (v/v) [20% (v/v) Solutol in 100 mM citrate buffer, pH 3, and dosed orally (PO) as a suspension once daily (QD) at 3 mg/kg, or BID at 5 mg/kg. Etoposide was prepared in 5% DMSO + 40% PEG300 + 5%Tween 80 + 50%dd water and dosed intraperitoneally QD at 1.25 mg/kg for 3 days for each week of the study. Olaparib was prepared in 10% HP-ß-CD in water and dosed intraperitoneally QD at 50 mg/kg. Tumor sizes and body weight were measured twice weekly. Tumor sizes were measured in two dimensions using a caliper, and the volume (mm^3^) was calculated using the formula: *V* = 0.5 *a* x *b*^2^ where *a* and *b* are the long and short diameters of the tumor, respectively.

### TCGA and DepMap data sources

We used publicly available data of patients with ovarian cancer from the TCGA to determine mutual exclusivity of *CCNE1* amplification and mutations in HR-related genes. To interrogate features with potential relationships to HR, we retrieved data from the DepMap 21Q4 public release (DepMap, Broad, 2021) of annotated cell lines from the CCLE. Specifically, fusion and copy number data were used to assess the landscape of gross chromosomal rearrangements across cancer types and tumor mutation burden estimated from mutation data and aneuploidy scores derived from ABSOLUTE copy number profiles were used to characterize differences in *CCNE1*-amplified versus non-amplified cell lines.

### Statistical analysis of mutual exclusivity in cancer patients and fusion events in cancer cell lines

Mutual exclusivity of *CCNE1* amplification and HR-related genes was analyzed in patients with ovarian cancer with mutation and copy number data provided by TCGA. *CCNE1*-amplified tumors had GISTIC-called amplifications while HR-mutated tumors had either missense, inframe, or truncation mutations identified by whole exome sequencing (25). The Fishers’ exact test was used to determine statistical significance (*P* ≤ 0.05), while odds ratios were calculated to assess the magnitude and direction of effect.

Fusions identified in DepMap 21Q4 release were called from RNA-sequencing data using the STAR-Fusion pipeline version 1.6.0 and filtered as described in DepMap documentation (26). To create a more robust call set, we further filtered out fusions with fusion fragments per million < 0.1 or a spanning fragment count of 0 with no long double anchor support. Copy number values greater than three were called as amplified, assuming diploidy. Differences in fusion distribution, tumor mutation burden, and aneuploidy were tested using the Wilcoxon rank-sum test (*P* ≤ 0.05). Differences in the proportion of cell lines were tested using the Fishers’ exact test (*P* ≤ 0.05).

All statistical analyses were performed in R 3.6.3.

## RESULTS

### *CCNE1-*amplified ovarian cancers have a high incidence of chromosomal fusion events

**Figure 1.**
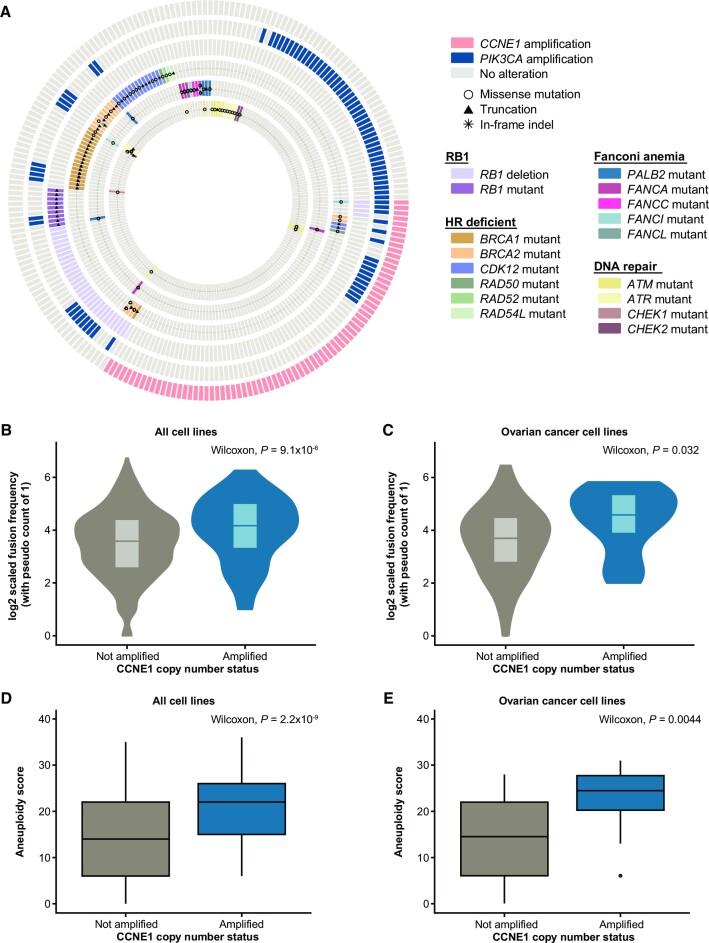
*CCNE1*-amplified cancer cell lines are prone to GCR. (**A**) Co-occurrence of *CCNE1*-amplification and somatic mutations in genes of interest in patients with ovarian cancer included in The Cancer Genome Atlas (n = 224). Each ring represents a gene or gene category (outer to inner: CCNE1, PIK3CA, RB1, HR deficient, Fanconi anemia-related genes, DNA repair genes, Holliday junction genes, replication fork genes). Each row of bars across the rings represents a patient with *CCNE1*-amplification and/or a somatic mutation in a gene of interest. The color of the bar specifies the gene being mutated; symbols within the bar represent the type of mutation. Patients with ovarian cancer with no amplifications or mutations in listed genes are not shown. (**B**) Distribution of fusion events in *CCNE1*-amplified and non-amplified cancer cell lines (*n* = 1371) (Wilcoxon Rank Sum Test, *P* = 9.1E-06). (**C**) Distribution of fusion events in *CCNE1*-amplified and non-amplified ovarian cancer cell lines (*n* = 64) (Wilcoxon Rank Sum Test, *P* = 0.032). (**D**) Difference in aneuploidy scores in *CCNE1-*amplified and non-amplified cancer cell lines (*n* = 942) (Wilcoxon Rank Sum Test, *P* = 2.2E-09). For each box plot, the center line represents the median aneuploidy score of each cell line category. The upper and lower boundary of box plots represent the 75th percentile and 25th percentile of aneuploidy score, respectively. The extreme ends of the line are the minimum and maximum values, excluding outliers. Any outliers are represented by a solid black point. (**E**) Difference in aneuploidy scores in *CCNE1*-amplified and non-amplified ovarian cancer cell lines (*n* = 48) (Wilcoxon Rank Sum Test, *P* = 0.0044). For each box plot, the center line represents the median aneuploidy score of each cell line category. The upper and lower boundary of box plots represent the 75th percentile and 25th percentile of aneuploidy score, respectively. The extreme ends of the line are the minimum and maximum values, excluding outliers. Any outliers are represented by a solid black point.

Co-occurrence of *CCNE1* amplification with *RB1* loss and somatic mutations in genes involved in HR, Fanconi anemia (FA), and DNA repair was assessed using patient data from The Cancer Genome Atlas (TCGA). In agreement with previous analyses, *CCNE1* amplification has a tendency toward mutual exclusivity with *RB1* loss and putative driver mutations in the HR and FA pathways (4) (Figure 1A, Supplementary Table S1). We also analyzed *PIK3CA*, a frequently amplified oncogene in ovarian cancer, and observed that *PIK3CA* and *CCNE1* amplification are not mutually exclusive (Supplementary Table S1). Because *CCNE1* amplification is not generally mutually exclusive with other oncogenic alterations, such as *PIK3CA* amplification, the mutual exclusivity between *CCNE1* amplification and HR may be governed by specific molecular mechanisms.

Because *CCNE1* amplification induces oncogenic stress and DSBs that can lead to allelic imbalances (10,27), we assessed gross chromosomal rearrangements (GCRs) in *CCNE1*-amplified cell lines versus non-amplified cell lines using data from the Cancer Cell Line Encyclopedia (CCLE). Across all indications, we found that *CCNE1*-amplified lines are more prone to chromosomal fusions than non-amplified lines (*P* = 9.1 × 10^−6^) (Figure 1B), and ovarian *CCNE1*-amplified cell lines showed the same trend compared with non-amplified cell lines (*P* = 0.032) (Figure 1C). Furthermore, these fusions are more likely to occur at cyclin E1-induced fragile sites (Supplementary Figure S1A,B) (28). We also observed that *CCNE1*-amplified cells tend to have higher levels of aneuploidy compared with non-amplified cell lines across tumor types (*P* = 2.2 × 10^−9^) (Figure 1D), and in ovarian cancer cell lines specifically (*P* = 0.0044) (Figure 1E). This observation is consistent with the role of cyclin E1 in endoreplication during development (29–31). *CCNE1*-amplified cells, however, do not have higher mutational burden compared with non-amplified cells (Supplementary Figure S1C, D). Taken together, these data support that *CCNE1*-amplified cell lines may be encountering barriers to replication in *CCNE1*-induced fragile sites, leading to chromosomal fusion events.

### *CCNE1*-amplified cells require CDK2 for CHK1 signaling, and HR for replication restart

**Figure 2.**
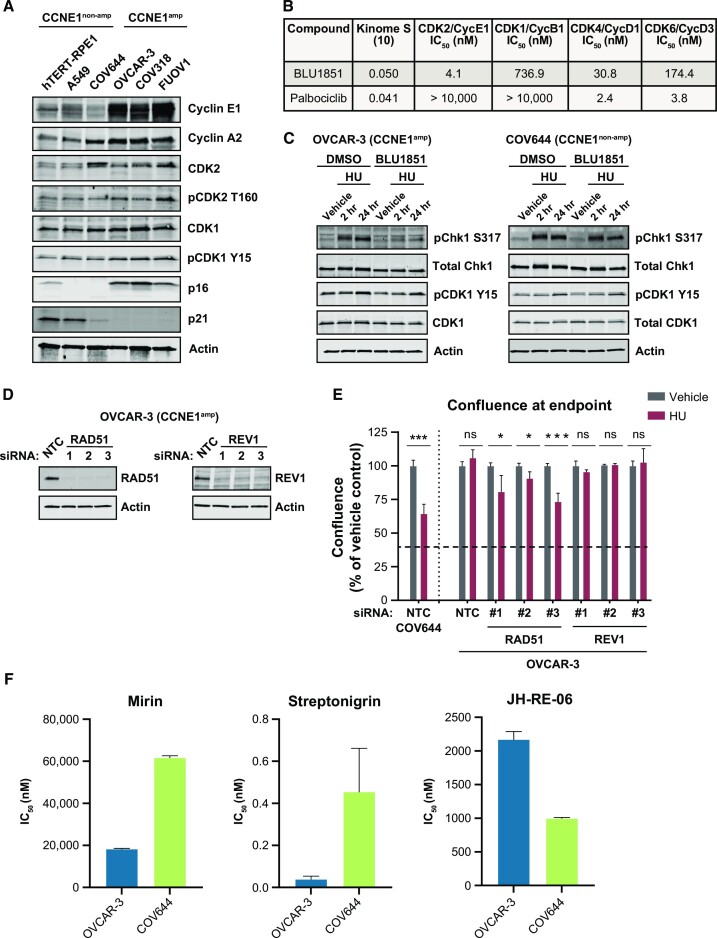
Replication fork stall signaling is dependent on CDK2, HR in *CCNE1*-amplified cells. (**A**) Western blot analysis of indicated antibodies in panel of *CCNE1*-amplified versus non-amplified cell lines. (**B**) Fraction of kinases with <10% of control at 3 μM among all the kinases tested (*n* = 468), measured by KINOMEscan platform, and IC_50_ (nM) of indicated compounds in ATP-competition caliper enzyme assay run at 1 mM ATP. (**C**) Western blot analysis of indicated antibodies in OVCAR-3 and COV644 cells. Cells were treated with BLU1851 (100 nM) for 24 h prior to addition of HU at indicated time points. (**D**) Western blot analysis of indicated antibodies. OVCAR-3 cells were transfected with non-targeting siRNA (NTC), RAD51 siRNA, or REV1 siRNA, and collected 48 h later. (**E**) Cell confluency at experimental endpoint. Confluence was measured by IncuCyte live cell imaging and values were determined using an algorithm to detect cells from phase-contrast images. Cells were transfected with siRNA 24 h before addition of HU (2 mM) for 24 h. After the HU was washed out, cells were transfected again with indicated siRNAs and allowed to incubate for 5 additional days. HU-treated groups were normalized to vehicle-treated groups for each siRNA at the endpoint. The dotted line represents confluency of cells treated with constant HU exposure over the time course of the experiment. Error bars represent standard deviation (SD) in duplicate experiments. Statistical analysis: Student's *t*-test, * = *P* < 0.05, ** = *P* < 0.01, *** = *P* < 0.001, **** = *P* < 0.0001, ns = not significant. (**F**) Proliferative IC_50_ (nM) of indicated compounds. Cells were treated with a 10-point dose response of indicated compounds and incubated for 5 days before CyQuant assay was performed. Error bars represent SD of at least duplicate independent experiments.

To choose model cell lines to interrogate the relationship between *CCNE1* amplification and HR, we measured protein levels of key cell cycle regulators in a panel of HR-proficient *CCNE1*-amplified and *CCNE1* non-amplified cells (Figure 2A, Supplementary Table S2). We chose OVCAR-3, FUOV1 and COV318 to represent ovarian cell lines with amplification of *CCNE1*, and COV644 to represent ovarian cell lines with two copies of *CCNE1*. We also included a diploid, immortalized cell line, hTERT-RPE1, to interrogate a non-cancerous cell line with an intact cell cycle (32), and A549, a cell line previously reported to be dependent on CDK1 for HR (Supplementary Table S2) (19). As expected, *CCNE1*-amplified cell lines had increased levels of cyclin E1 protein (Figure 2A), consistent with patient data from the TCGA (Supplementary Figure S2A). *CCNE1*-amplified cell lines also had markers of active CDK2 (elevated phosphorylation at T160, elevated levels of cyclin A2, low p21) (Figure 2A). Notably, *CCNE1*-amplified cell lines also had high levels of p16, a negative regulator of cyclin-dependent kinase 4/6 (CDK4/6) (33), and elevated levels of pCDK1 Y15, an inhibitory phosphorylation site on CDK1 (Figure 2A) (34). The protein levels of these markers indicate that CDK2 may be the predominantly active CDK in this setting.

Because the response to replication fork stalling is controlled mainly by the CDK2-ATRIP-ATR-CHK1 axis (35), we utilized a selective CDK2 inhibitor, BLU1851 (Supplementary Figure S2B), to interrogate this signaling cascade in *CCNE1*-amplified cells. BLU1851 is a potent and specific CDK2 inhibitor with limited off-target activity against the entire kinome, including other related CDK family members, such as CDK1 (Figure 2B, Supplementary Figure S2C–H, Tables S3A–C) (36). To keep concentrations in a selective range over the key off-target CDK1 for our mechanistic experiments, we tested BLU1851 in cellular phosphorylation assays to measure CDK2 and CDK1 activity. We measured phosphorylation of Rb in OVCAR-3 as a CDK2 readout (37), and phosphorylation of lamin A/C as a CDK1 readout (38) and found that there is a large window between CDK2 and CDK1 activity (Supplementary Figure S2F,G). Lastly, because there was some activity on CDK4 in the enzyme assay (Supplementary Figure S2D, Table S3A), we tested the anti-proliferative effect of BLU1851 on a CDK4-dependent cell line, MCF-7, and calculated an average IC_50_ > 1 uM, suggesting BLU1851 does not potently target CDK4 (Supplementary Figure S2H, Table S3C). Based on these data, we chose CDK2-selective concentrations of BLU1851 in our studies.

Given the overall chromosomal instability and markers of hyperactive CDK2 observed in *CCNE1*-amplified cells, we hypothesized that these cells may be especially reliant on CDK2-CHK1 signaling in response to stalled or collapsed replication forks. We therefore measured CHK1 activation via S317 phosphorylation in both OVCAR-3 and COV644 cells after 2 and 24 h of hydroxyurea (HU) treatment to stall or collapse replication forks, respectively, in the presence or absence of BLU1851, at a concentration below the proliferative half-maximal inhibitory concentration (IC_50_) in OVCAR-3 (Supplementary Table S3C) to limit the effects of the compound on the cell cycle. While CHK1 signaling was expectedly activated in both cell lines following HU treatment, CDK2 inhibition suppressed pCHK1 S317 to a greater extent in OVCAR-3 cells compared with COV644 cells (Figure 2C, Supplementary Figure S2I). BLU1851 treatment also inhibited pCHK1 S317 in FUOV1 and COV318 to a greater extent than in hTERT-RPE1 or A549 cells (Supplementary Figure S2J). These data support a more prominent role for CDK2 in replication fork maintenance via CHK1 in *CCNE1*-amplified cells compared with non-amplified cells.

As an avenue for fork restart or repair, cells can use HR-mediated mechanisms such as DSB repair after nucleolytic cleavage of a regressed fork, template switching, and gap repair, or they can utilize alternative, more mutagenic pathways such as translesion synthesis (TLS) (39). To first validate that we could prevent proliferative recovery from replication fork collapse via HU, we treated OVCAR-3 cells with the CHK1 inhibitor, rabusertib, at a concentration that would not inhibit proliferation on its own followed by a 24-h pulse of HU (40) (Supplementary Figure S2K). Indeed, CHK1 inhibition suppressed the ability of OVCAR-3 cells to proliferate following HU treatment (Supplementary Figure S2K). We next utilized this assay to measure the ability of OVCAR-3 and COV644 cells to resume proliferation after HU-induced replication fork collapse by using small interfering RNA to knockdown the HR factor RAD51, or the TLS factor, REV1. COV644 cells were overall less efficient at recovering from HU, while OVCAR-3 cells were able to resume proliferation and reach the confluence of vehicle-treated controls (Figure 2E). However, when we depleted RAD51 in OVCAR-3 cells, we observed that cells were less efficient at proliferating following HU treatment compared with non-targeting control, while REV1 depletion had no significant effect (Figure 2D, E, Supplementary Figure S2L). In contrast, neither RAD51 nor REV1 depletion in COV644 cells worsened the recovery from HU compared with non-targeting control, albeit with less efficient knockdown compared to OVCAR-3 (Supplementary Figure S2M,N). These data support the hypothesis that *CCNE1*-amplified cells rely, at least in part, on HR-mediated mechanisms to repair collapsed replication forks, while non-amplified cells may utilize multiple repair pathways to compensate for the loss of one pathway.

Since *CCNE1*-overexpressing cells incur endogenously higher rates of DNA damage through accumulation of DSBs (10), and RAD51 knockdown impaired the ability of OVCAR-3 cells to recover from induced replication fork collapse, we reasoned that OVCAR-3 cells would be more sensitive to inhibitors that disrupt the HR pathway compared with COV644 cells if they do indeed utilize HR to combat replication stress. To test this, we treated cells with mirin, which inhibits the exonuclease activity of MRE11, streptonigrin, which inhibits RAD54 ATPase activity, and JH-RE-06, a REV1-REV7 protein-protein interaction inhibitor, to model inhibition of early-stage resection, later stage HR, and TLS, respectively (41–43). OVCAR-3 cells were more sensitive to both mirin and streptonigrin compared with COV644 cells, while COV644 cells were more sensitive to JH-RE-06 (Figure 2F). These data suggest that OVCAR-3 cells require HR, but not TLS, for proliferation to a greater extent than COV644 cells.

### Cyclin E1 is present at the site of stalled replication forks in *CCNE1*-amplified cells

**Figure 3.**
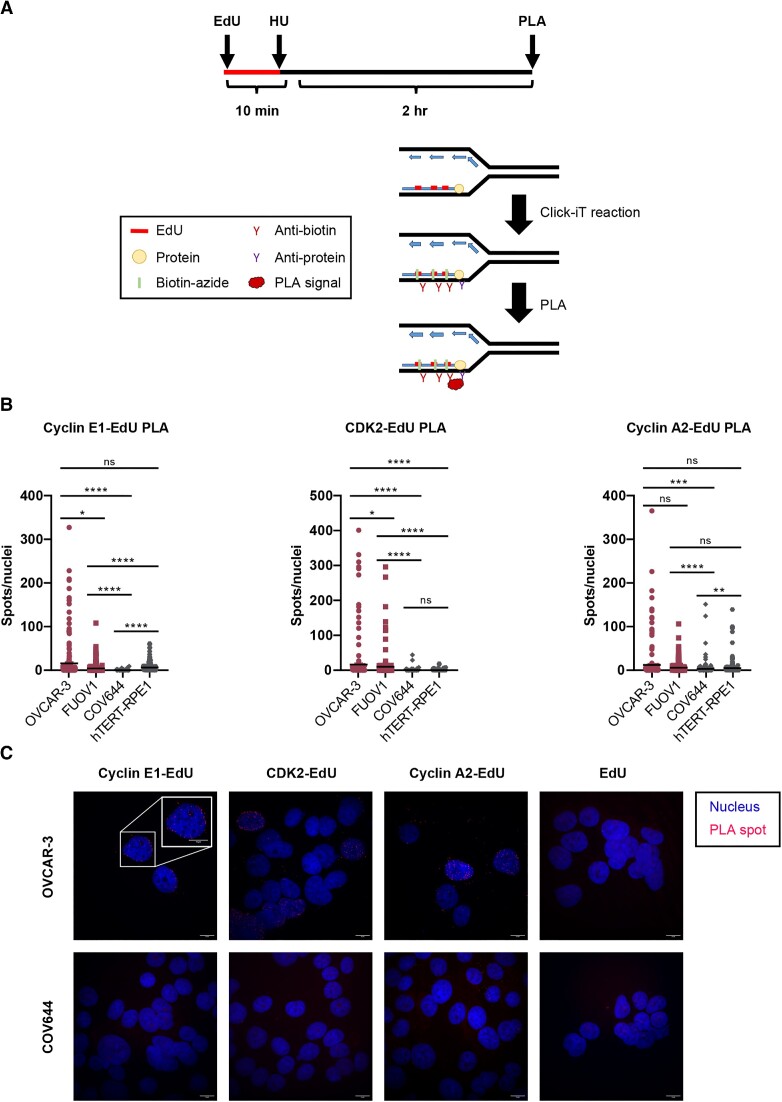
CDK2 and cyclin E1 are recruited to stalled replication forks in *CCNE1*-amplified cells. (**A**) Schematic of protein recruitment to stalled replication forks using EdU-proximity ligation assay (PLA). PLA was performed using anti-biotin with either anti-CDK2, anti-cyclin E1, or anti-cyclin A2 antibodies. The negative control excluded one primary antibody. (**B**) The graphs shows the mean number of PLA spots per nucleus in each indicated PLA assay. Bars represent the mean (NS = not significant, * = *P* < 0.05, ** = *P* < 0.01, *** = *P* < 0.001, **** *P* < 0.0001, Mann–Whitney test; *n* = at least 100 cells/experiment). (**C**) Representative images of PLA for each noted interaction in OVCAR-3 and COV644 cells. PLA spots are shown in red and nuclei are shown in blue.

Since *CCNE1-*amplified cells are dependent on CDK2 for replication fork stall signaling, we measured the physical presence of CDK2 and its canonical cyclin partners, cyclin E1 and cyclin A2, at the site of stalled forks in two *CCNE1*-amplified lines, OVCAR-3 and FUOV1, and two non-amplified lines, COV318 and hTERT-RPE1. We used a proximity ligation assay (44) to detect the presence of cyclin E1, cyclin A2 and CDK2 at 5′-ethylene-2′-deoxyuridine (EdU) labeled nascent DNA stalled by HU (Figure 3A) (45). Surprisingly, OVCAR-3 and FUOV1 cells not only had significantly more cyclin E1 at the site of stalled replication forks compared with COV644 cells, but also significantly more CDK2 and cyclin A2 (Figure 3B, C, Supplementary Figures S3A, B). The physical proximity of CDK2 along with its canonical cyclin partners suggests that catalytically active CDK2 has a role directly at the site of the stalled fork. Additionally, the detection of cyclin E1 at stalled forks in *CCNE1*-amplified cell lines suggests that cyclin E1 could be participating in non-canonical replication activities outside of its known functions in pre-replication complex assembly (46). While COV644 cells had less CDK2 and cyclins present at stalled forks compared with OVCAR-3 and FUOV1 cells, hTERT-RPE1 did not display significant differences in cyclin A2 levels, but did have significantly fewer instances of CDK2 recruitment and trended toward less cyclin E1 levels (Figure 3B, Supplementary Figure S3A). Overall, these data suggest that CDK2 is acting physically at the site of stalled replication forks in the *CCNE1*-amplified setting.

### CDK2 regulates homologous recombination in *CCNE1*-amplified ovarian cells

**Figure 4.**
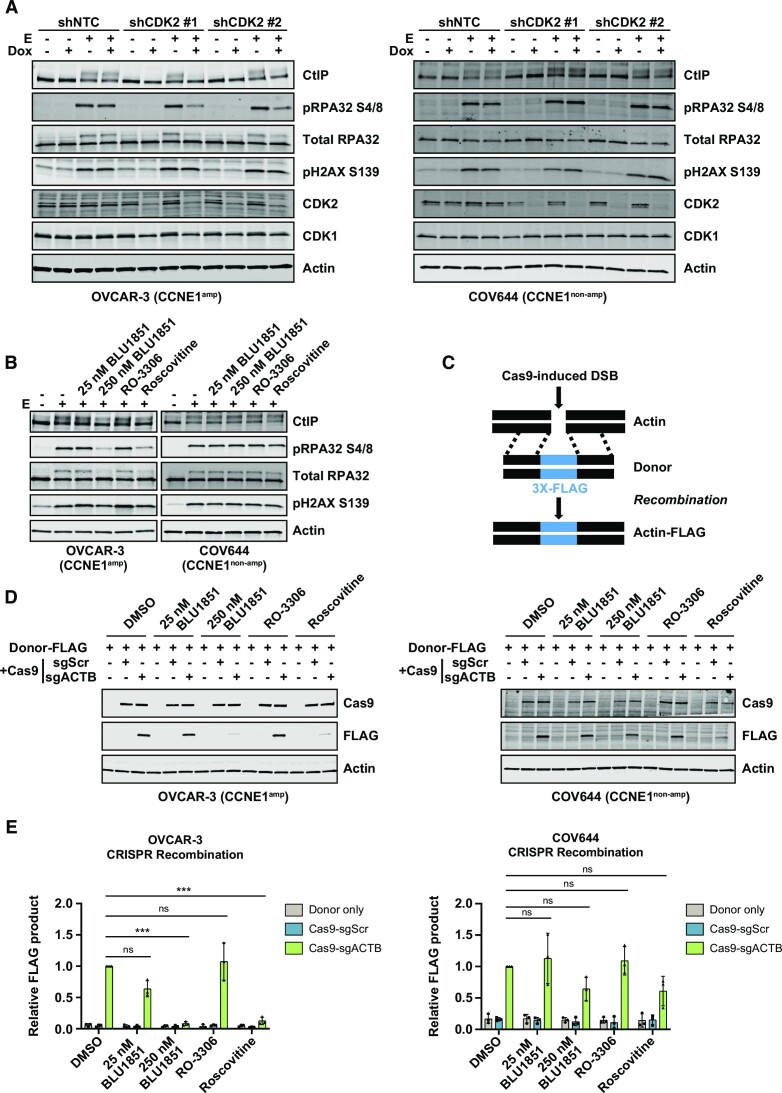
CDK2 regulates HR in *CCNE1*-amplified cell lines. (**A**) Western blot analysis with the indicated antibodies. Small hairpin RNA were induced by doxycycline (dox) for 96 h prior to etoposide (E) treatment at 50 μM for 2 h. (**B**) Western blot analysis with the indicated antibodies. OVCAR-3 or COV644 cells were treated with indicated 25 or 250 nM BLU1851, 1 μM RO-3306 or 10 μM roscovitine for 24 h before being treated with 50 μM etoposide (E) for 2 h. (**C**) Schematic of CRISPR recombination assay. Cas9 induces a double stranded break upstream of the actin stop codon. A donor plasmid with homology on either side of the cut site with an inserted 3X-FLAG tag is provided. (**D**) Western blot analysis with indicated antibodies. OVCAR-3 or COV644 cells were treated with 25 nM or 250 nM BLU1851, 1 μM RO-3306, or 10 μM roscovitine. Contemporaneously, cells were transfected with a 3X-FLAG donor plasmid plus a plasmid containing Cas9 and a single guide RNA (sgRNA), scramble control (sgScr), or a sgRNA targeting beta-actin (sgACTB). Cells were harvested 72 h later. (**E**) Quantification of recombination product in (**D**) normalized to actin and to the DMSO sgACTB sample. Error bars represent SD in three independent experiments. Statistical analysis: One-way analysis of variance (ANOVA), * = *P* < 0.05, ** = *P* < 0.01, *** = *P* < 0.001, **** = *P* < 0.0001, ns = not significant.

Because *CCNE1*-amplified cells require CDK2 for stalled fork signaling via CHK1 (Figure 2C, Supplementary Figure S2H), and may restart forks through HR (Figure 2E), we hypothesized that CDK2 may be the exclusive kinase regulating HR in tumors with this genetic background. To test this, we engineered stable cell lines expressing doxycycline-inducible CDK2 and CDK1 short hairpin RNA (shRNA) in a panel of *CCNE1*-amplified and non-amplified cell lines. After knockdown of CDK2 or CDK1 in each line, we treated cells acutely with the topoisomerase II inhibitor, etoposide, to induce DSBs (47). We confirmed induction of DNA damage by pH2AX S139, and examined HR signaling via CtIP bandshift and pRPA S4/8 levels as a measure of DNA end resection (48,49) (Figure 4A, Supplementary Figures S4A-F). Strikingly, CDK2 depletion had little effect on HR signaling in the *CCNE1* non-amplified cell lines COV644, hTERT-RPE1, and A549, but suppressed HR signaling in the *CCNE1*-amplified cell lines OVCAR-3, COV318, FUOV1 (Figure 4A, Supplementary Figures S4A–F). CDK1 depletion had little impact on HR signaling in any cell line irrespective of *CCNE1* copy number (Supplementary Figures S4B-F). Consistent with the data from inducible CDK2 knockdown, the CDK2 inhibitor BLU1851 suppressed HR signaling as measured by the CtIP bandshift and pRPA S4/8 in *CCNE1*-amplified cell lines in a dose-dependent manner but had little effect in non-amplified cell lines (Figure 4B, Supplementary Figure S4G, H). While the selective CDK1 inhibitor, RO-3306 (50), had only a small effect in some cell lines, the CDK1/2 dual inhibitor, roscovitine (51), suppressed HR signaling in most cell lines tested (Figure 4B, Supplementary Figure S4G, H). Taken together, these data suggest that CDK2 may be regulating HR non-redundantly in *CCNE1*-amplified cells.

To test if CDK2 inhibition can inhibit HR in *CCNE1*-amplified cells, we employed a CRISPR/Cas9-based recombination assay to induce a targeted DSB upstream of the beta-actin stop codon and provided a donor template with an inserted 3X-FLAG reporter (52) (Figure 4C). As expected from the signaling data (Figure 4B, Supplementary Figure S4H), recombination was suppressed in OVCAR-3 and FUOV1 cells but not in COV644 or hTERT-RPE1 cells after treatment with BLU1851 (Figure 4D, E, Supplementary Figure S4I). Treatment with roscovitine, in contrast, inhibited recombination in all cell lines (Figure 4D, E, Supplementary Figure S4I). These data suggest that, in the *CCNE1*-amplified setting, CDK2 is uniquely required for recombination, while CDK1 and CDK2 may have redundant roles in non-amplified cells.

Because repair pathway choice is in part dictated by cell cycle phase (53), and CDK2 inhibition arrests cells at the G1/S boundary in *CCNE1*-amplified cells (Supplementary Figure S4J), we tested if the suppression of HR signaling after CDK2 inhibition in *CCNE1*-amplified cells was a consequence of cell cycle arrest or if CDK2 was directly acting in the HR pathway. We compared cells arrested at G1/S with double thymidine block or with BLU1851 treatment. While both methods arrested cells in G1 as measured by flow cytometry (Supplementary Figure S4K), only treatment with the CDK2 inhibitor suppressed HR signaling after etoposide treatment (Supplementary Figure S4L). These data support that CDK2 is functioning in HR directly and the observed effects are not simply a consequence of cell cycle arrest.

### CDK2 inhibition sensitizes *CCNE1*-amplifed cells to DNA damaging agents *in vitro* and *in vivo*

**Figure 5.**
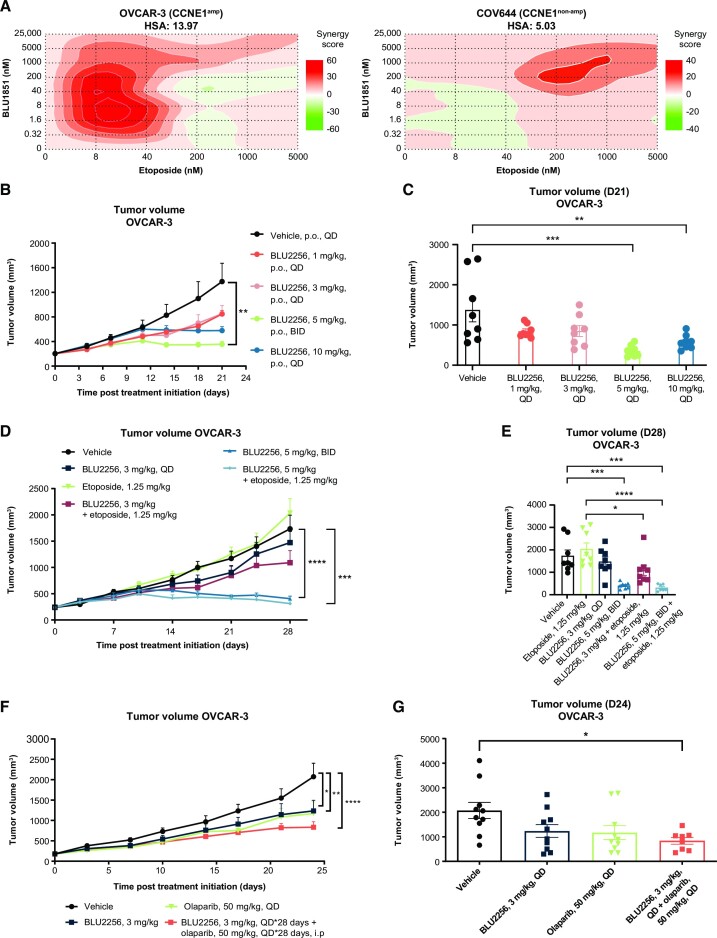
CDK2 inhibition sensitizes *CCNE1*-amplified cells to DNA damaging agents *in vitro* and *in vivo*. (**A**) Synergy score heat maps in OVCAR-3 or COV644 cells after combination treatment with BLU1851 and etoposide. Highest single agent (HSA) synergy model was used to calculate the synergy scores in Synergy Finder. A score ←10 is indicative of antagonism, a score between –10 and 10 is indicative of additivity, and a score >10 is indicative of synergy. Data are representative of at least four independent experiments. (**B**) Tumor volume over time in mice harboring OVCAR-3 xenografts treated with vehicle or BLU2256 at indicated doses QD for 3 weeks. *N* = 8/treatment group. A two-way ANOVA repeated measures (RM) was performed on day 21 to compare the tumor volume between treatment groups and vehicle groups. Comparisons between groups were carried out with Tukey multiple comparison test. *P* < 0.05 was considered to be statistically significant. * = *P* < 0.05, ** = *P* < 0.01, *** = *P* < 0.001, **** = *P* < 0.0001. (**C**) Tumor volume at day 21 shown in (B). A one-way ANOVA RM was performed to compare the tumor volume between treatment groups and vehicle groups. Comparisons between groups were carried out with Tukey multiple comparison test. *P* < 0.05 was considered to be statistically significant. * = *P* < 0.05, ** = *P* < 0.01, *** = *P* < 0.001, **** = *P* < 0.0001. (**D**) Tumor volume over time in mice harboring OVCAR-3 xenografts treated with vehicle, BLU2256 at indicated doses, Etoposide at 1.25 mg/kg (3 days on, 4 days off). *N* = 8/treatment group. A two-way ANOVA RM was performed on day 28 to compare the tumor volume between treatment groups and vehicle groups. Comparisons between groups were carried out with Tukey multiple comparison test. *P* < 0.05 was considered to be statistically significant. * = *P* < 0.05, ** = *P* < 0.01, *** = *P* < 0.001, **** = *P* < 0.0001. (**E**) Tumor volume at day 28 shown in (D). A one-way ANOVA RM was performed to compare the tumor volume between treatment groups and vehicle groups. Comparisons between groups were carried out with Tukey multiple comparison test. *P* < 0.05 was considered to be statistically significant. * = *P* < 0.05, ** = *P* < 0.01, *** = *P* < 0.001, **** = *P* < 0.0001. (**F**) Tumor volume over time in mice harboring OVCAR-3 xenografts treated with vehicle, BLU2256 at indicated doses, olaparib at 50 mg/kg QD. *N* = 8–10/treatment group. A two-way ANOVA RM was performed on day 24 to compare the tumor volume between treatment groups and vehicle groups. Comparisons between groups were carried out with Tukey multiple comparison test. *P* < 0.05 was considered to be statistically significant. * = *P* < 0.05, ** = *P* < 0.01, *** = *P* < 0.001, **** = *P* < 0.0001. **(G)** Tumor volume at day 24 shown in (F). A one-way ANOVA RM was performed to compare the tumor volume between treatment groups and vehicle groups. Comparisons between groups were carried out with Tukey multiple comparison test. *P* < 0.05 was considered to be statistically significant. * = *P* < 0.05, ** = *P* < 0.01, *** = *P* < 0.001, **** = *P* < 0.0001.

We have shown that CDK2 loss or inhibition suppresses HR signaling and recombination, and thus reasoned that combining CDK2 inhibition with a chemotherapy that induces DSBs, such as etoposide, should be synergistic. We tested a dose matrix of BLU1851 and etoposide in OVCAR-3 and COV644 cells *in vitro* and observed that the combination was synergistic in OVCAR-3 cells (highest single agent [HSA] = 13.97), but not in COV644 cells (HSA = 5.03) (Figure 5A). Notably, the strongest levels of synergy in OVCAR-3 cells were observed at low concentrations of both agents, below the proliferative IC_50_. These same doses in COV644 cells trend toward antagonism. The combination effect at low doses in OVCAR-3 cells suggests that the underlying mechanism is based on the DNA damage function, rather than the proliferative function, of CDK2. These data support the hypothesis that CDK2 regulates HR in *CCNE1*-amplified cells.

To test if a CDK2 inhibitor in combination with etoposide is anti-tumorigenic *in vivo*, we used the OVCAR-3 xenograft, a model previously used to validate CDK2 as an oncogenic driver in *CCNE1*-amplified ovarian cancer (54). In these studies, we used BLU2256, a second selective CDK2 inhibitor that suppresses HR signaling and recombination in *CCNE1*-amplified cells. BLU2256 is structurally related to BLU1851 and showed similar *in vitro* phenotypes, but with superior pharmacokinetics, making it more suitable than BLU1851 for *in vivo* studies (Supplementary Figures S5A–J, Tables S4A–C) (36). BLU2256 demonstrated dose-dependent tumor growth inhibition in OVCAR-3 xenografts without a change in body weight (Figure 5B, C, Supplementary Figure S5K). We determined on-target activity of this agent by measuring the inhibition of RB1 phosphorylation, which was dose and time-dependent (Supplementary Figures S5L,M). Based on the dose-titration data, we determined that a 3 mg/kg QD dose was sub-efficacious with minor tumor growth inhibition, while 5 mg/kg twice daily (BID) was an efficacious dose, inducing tumor stasis. We tested both doses of BLU2256 in combination with a low-dose of etoposide (1.25 mg/kg) and observed there is only a benefit of combination treatment when CDK2 is inhibited sub-optimally at 3 mg/kg QD, consistent with the *in vitro* data (Figure 5D, E, Supplementary Figure S5N).

Because HR impairment is synthetically lethal with PARP inhibition (2,3) we reasoned that a CDK2 inhibitor, in combination with a PARP inhibitor should show greater anti-tumor activity compared to single agent. To test this, we treated mice harboring the OVCAR-3 xenograft model with BLU2256 at 3 mg/kg QD, olaparib at 50 mg/kg QD, or the combination. We observed that the combination of BLU2256 and olaparib had better anti-tumor activity compared to the single agent treatments (Figure 5F,G, Supplementary Figure S5O). These data support a mechanism by which CDK2 inhibition suppresses HR in *CCNE1*-amplfied cells and induces an anti-tumorigenic phenotype when in combination with DNA damaging agents.

## DISCUSSION

This study provides a mechanism to explain the mutual exclusivity between *CCNE1* amplification and HRD in HGSOC. We propose that *CCNE1*-amplified ovarian cancer cells rely on HR to resume replication after fork collapse. We also measured high levels of CDK2, cyclin E1, and cyclin A2 protein at the site of stalled replication forks in *CCNE1*-amplified cells which may indicate direct CDK2 catalytic activity at stalled forks. Lastly, we demonstrate that CDK2 is non-redundantly regulating stalled/collapsed replication fork signaling via CHK1 and HR following induced DNA damage in *CCNE1*-amplified cells. Inhibition of CDK2 in combination with etoposide showed better efficacy compared with a single agent at low doses *in vitro* and *in vivo*. This work suggests CDK2 inhibitors in combination with DNA damaging agents may have added clinical benefit.

While previous studies have noted the mutual exclusivity between *CCNE1*-amplification and HRD (4), the molecular process governing this relationship has not been adequately detailed. Our proposed mechanism that *CCNE1*-amplified cells utilize HR to combat replication stress is consistent with reports that HR genes are upregulated following *CCNE1* overexpression in fallopian tube secretory epithelial cells, the presumed cell of origin for HGSOC (55). A similar mechanism may be apparent in cancer cell lines that grow despite the constant replication stress imposed by high cyclin E1 levels. The frequency of replication fork collapse in *CCNE1* overexpressing human fibroblast cells reveals unique fragile sites in the genome, many of which are instability hot spots in cancer and are prone to rearrangement (28). Consistent with this finding, our GCR analysis revealed that *CCNE1*-amplified cells are more likely to have chromosomal fusion events overall, and these fusion events are more likely to occur in *CCNE1*-induced fragile sites. The higher levels of aneuploidy observed in *CCNE1*-amplified cells are also consistent with the hypothesis that replication stress imposed by *CCNE1*-levels induces GCR. In contrast, we did not observe a higher mutational burden in *CCNE1*-amplified cells, which would be associated with more error-prone processes, such as NHEJ or replication restart through TLS. These data support a hypothesis that *CCNE1*-amplified cells have barriers to replication and may be using HR as a method to resume replication at the site of replication fork stall/collapse.

The role of CDK2 in this process has been less clear, as a recent report suggested that unrestrained CDK2 activity can promote excessive fork degradation (56), despite known roles in maintaining genome integrity (57,58). A major limitation and potential confounding factor in past studies is the use of non-selective kinase inhibitors to interrogate CDK functions, or the use of a CDK2 analog-sensitive mutant that has impaired cyclin binding and thus low kinase activity (59). Here, we utilized specific and potent CDK2 inhibitors with selectivity not only over other CDK family members but also the entire kinome, to more precisely assess how CDK2 is promoting repair signaling in *CCNE1*-amplified cells. Our study suggests CDK2 is required in *CCNE1*-amplified cells both for replication fork signaling, as well as HR but is dispensable in non-amplified cells. To our knowledge, this is the first report demonstrating exclusive dependence on CDK2 for DNA repair.

Prior to this study, neither cyclin A2 nor cyclin E1 had been detected at the site of stalled replication forks using methods such as isolation of proteins on nascent DNA (iPOND) (60), potentially because this technique is not suited for transient or low abundance interactions. The data presented here indicate active CDK2 is directly associating with stalled replication forks, especially in the *CCNE1*-amplified setting. In contrast, CDK2 does not appear to be a prime actor in replication fork maintenance in *CCNE1* non-amplified cell lines. The physical presence of CDK2, cyclin A2, and cyclin E1 at stalled replication forks in *CCNE1*-amplified cells may indicate these complexes are functioning to promote repair and restoration of the fork. Determining the precise function of CDK2-cyclin E1 or CDK2-cyclin A2 at the site of stalled forks in *CCNE1*-amplified cells is an area of future research.

Loss of CDK2 function can typically be compensated for by other CDK family members, especially by CDK1 (61,62). Indeed, in previous reports, CDK1 was identified as essential for HR and synergized with DNA damaging agents, while CDK2 was dispensable (19,24). The present study shows that CDK2 is essential for HR in the *CCNE1*-ampified context and selective inhibition of CDK2 at sub-efficacious doses can sensitize cells to DNA damaging agents. CDK2 is an attractive therapeutic target as a single agent in *CCNE1*-amplified cancers as cell lines and xenograft models harboring the amplification fail to proliferate after knockdown or catalytic inhibition of CDK2 (54). When treated in combination with etoposide or olaparib, we demonstrated added anti-tumor activity when CDK2 inhibition was sub-efficacious. Because PARP inhibitors have had a major clinical impact in HR-deficient tumors, inducing an HRD phenotype to reveal synthetic lethality with a PARP inhibitor is attractive (2,3). These findings will also be important for clinical applications if patients receiving CDK2 inhibitors need to dose reduce, or to limit potential drug-drug interactions when treated in combination. These data support a CDK2-driven HR mechanism in *CCNE1*-amplified HGSOC.

Our data suggest a model in which *CCNE1*-amplified cell lines rely on HR to repair collapsed replication forks. In addition to its role as a cell cycle regulator, CDK2 non-redundantly coordinates HR in this genetic context. While *CCNE1*-amplified cells have engaged HR as a survival mechanism to combat replication stress, inhibiting this process leaves them vulnerable to synthetic lethality with chemotherapies. As CDK2 inhibitors enter the clinic for HGSOC (clinicaltrials.gov: NCT04553133, NCT05252416), rational drug combinations could open additional clinical avenues and improve patient outcome.

## Supplementary Material

zcad039_Supplemental_File

## Data Availability

The data underlying this article will be shared on reasonable request to the corresponding author.

## Notes

Present address: Hsin-Jung Wu, Tango Therapeutics, Boston, MA 02215, USA.

Present address: Yoon Jong Choi, LifeMine Therapeutics, Cambridge, MA 02140, USA.
