# Supplementary material for: CDK2 regulates collapsed replication fork repair in CCNE1-amplified ovarian cancer cells via homologous recombination
Source: NAR Cancer. 2023 Jul 27;5(3):zcad039. doi: 10.1093/narcan/zcad039 (PMC10373114; doi:10.1093/narcan/zcad039)
Supplement: zcad039_Supplemental_File [file zcad039_supplemental_file.pdf]

CDK2 regulates collapsed replication fork repair in *CCNE1*-amplified ovarian cancer cells via homologous recombination

Victoria E. Brown[1,2,*], Sydney L. Moore[1,2], Maxine Chen[1], Nealia House[1], Philip Ramsden[1], Hsin-Jung Wu[1,3], Scott Ribich[1], Alexandra R. Grassian[1], and Yoon Jong Choi[1,4]

[1]Blueprint Medicines, Cambridge, MA 02139, USA

[2]Department of Biology, Tufts University, Medford, MA 02155, USA

[3]Present address: Tango Therapeutics, Boston, MA 02215, USA

[4]Present address: LifeMine Therapeutics, Cambridge, MA 02140, USA

SUPPLEMENTARY MATERIALS

MATERIALS 2

Cell lines 2

Antibodies 3

Critical commercial assays 4

Compounds 5

Plasmids 5

siRNA 5

sgRNA 6

shRNA 6

TABLES 7

Table S1. *CCNE1* amplification trends toward mutually exclusivity with HRD, FA, and DNA repair pathway mutations 7

Table S2. *CCNE1*, p53, BRCA status for model cell lines 8

Table S3. BLU1851 is a selective CDK2 inhibitor 9

Table S4. BLU2256 is a selective CDK2 inhibitor 10

SUPPLEMENTARY FIGURES 11

Figure S1. *CCNE1*-amplified ovarian cancer cell lines are prone to GCR 11

Figure S2. Replication fork stall signaling is dependent on CDK2, HR in *CCNE1*-amplified cells 12

Figure S3. CDK2 and cyclin E1 is recruited to stalled replication forks in *CCNE1*-amplified cells 16

Figure S4. CDK2 regulates HR in *CCNE1*-amplified cell lines 17

Figure S5. BLU2256 is a selective CDK2 inhibitor 22

MATERIALS

Cell lines

CELL LINE	SOURCE	CATALOG NUMBER
OVCAR-3	American Tissue Type Collection	HTB-161
hTERT-RPE1	American Tissue Type Collection	CRL-4000
A549	American Tissue Type Collection	CRL-185
COV644	Millipore Sigma	07071908
COV318	Millipore Sigma	07071903
FUOV1	DSMZ	ACC 444
MCF-7	American Tissue Type Collection	HTB-22

Antibodies

Western blot

ANTIBODY	SOURCE	CATALOG NUMBER
Cdk2	Santa Cruz Biotechnology	SC-6248, RRID:AB_627238
p16	Cell Signaling Technology	CST-80772, RRID:AB_2799960
Cyclin E1	Santa Cruz Biotechnology	SC-247, RRID:AB_627357
pRb S807/811	Cell Signaling Technology	CST-8516 , RRID:AB_11178658
Actin	Cell Signaling Technology	CST-8457, RRID:AB_10950489
p21	Cell Signaling Technology	CST-2947, RRID:AB_823586
Cyclin A2	Cell Signaling Technology	CST-4656, RRID:AB_2071958
CtIP	Cell Signaling Technology	CST-9201, RRID:AB_10828593
Actin	Cell Signaling Technology	CST-3700, RRID:AB_2242334
pH2A.X S139	Cell Signaling Technology	CST-9718, RRID:AB_2118009
pRPA S4/8	Bethyl	Bethyl #A300-245A, RRID:AB_210547
pCdk2 T160	Cell Signaling Technologies	CST-2561, RRID:AB_2078685
Cdk1	Santa Cruz Biotechnology	SC-54, RRID:AB_627224
pCdk1 Y15	Cell Signaling Technologies	CST-4539, RRID:AB_560953
pChk1 S317	Cell Signaling Technologies	CST-2344, RRID:AB_331488
Chk1	Cell Signaling Technologies	CST-2360, RRID:AB_2080320
RPA32	Cell Signaling Technologies	CST-2208 RRID:AB_2238543
Tubulin	Sigma-Aldrich	T9026, RRID:AB_477593
Rad51	Abcam	Ab1333524, RRID: AB_2722613
Rev1	Santa Cruz Biotechnology	SC-393022; RRID: Ab_2885169
Donkey anti-mouse	LI-COR	926-32212, RRID:AB_621847
Donkey anti-rabbit	LI-COR	926-32213,

		RRID:AB_621848
Goat anti-mouse	LI-COR	926-68070, RRID:AB_10956588
Goat anti-rabbit	LI-COR	926-68071, RRID:AB_10956166
Goat anti-rat	LI-COR	926-68076, RRID:AB_10956590

Phospho-Lamin S22 AlphaLISA

ANTIBODY	SOURCE	CATALOG NUMBER
pLamin S22	Cell Signaling Technologies	13448, RRID:AB_2798221
Total Lamin	Santa Cruz Biotechnology	SC-7292, RRID:AB_627875

Proximity ligation assay

ANTIBODY	SOURCE	CATALOG NUMBER
Biotin	Cell Signaling Technologies	5597, RRID:AB_10828011
Biotin	Sigma-Aldrich	SAB4200680
Cdk2	Santa Cruz Biotechnology	SC-6248; RRID: AB_627238
Cyclin E1	Sigma-Aldrich	SAB1400044, RRID:AB_184738
Cyclin A2	Santa Cruz Biotechnology	SC-271682, RRID:AB_10709300

Critical commercial assays

ASSAY	SOURCE	CATALOG NUMBER
Click-iT EdU Alexa Fluor 488 Flow Cytometry Assay Kit	Thermo Fisher Scientific	C140424
FxCycle Violet	Thermo Fisher Scientific	F10347
CyQuant Direct Cell Proliferation Assay	Thermo Fisher Scientific	C35011
Phospho-Rb (Thr821/826) AlphaLISA SureFire Ultra	Perkin Elmer	ALSU-PRB-B500

Compounds

ASSAY	SOURCE	CATALOG NUMBER
BLU1851	Blueprint Medicines	N/A
BLU2256	Blueprint Medicines	N/A
RO-3306	Selleck Chemicals	S7747
Roscovitine	Selleck Chemicals	S1153
Etoposide	Millipore Sigma	E1383
Hydroxyurea	Millipore Sigma	H8627
Rabusertib	Selleck Chemicals	S2626
Mirin	Selleck Chemicals	S8096
Streptonigrin	Sigma Aldrich	S1014
JH-RE-06	Selleck Chemicals	S8850
DMSO	Thermo Fisher Scientific	D12345
Doxycycline	Takara	631311

Plasmids

VECTOR	SOURCE
pBluescript II SK+	Genscript
pLentiCRISPRV2	Genscript
SMARTvector inducible shRNA	Horizon Discovery

siRNA

TARGET	SOURCE	CATALOG NUMBER
NTC	Horizon Discovery	D-001810-01-20
RAD51 #1	Horizon Discovery	J-003530-10
RAD51 #2	Horizon Discovery	J-003530-11
RAD51 #3	Horizon Discovery	J-003530-12
REV1 #1	Horizon Discovery	J-008234-07
REV1 #2	Horizon Discovery	J-008234-08
REV1 #3	Horizon Discovery	J-008234-05

sgRNA

TARGET	SOURCE	SEQUENCE
sgScramble	GenScript	5′-CCTGGGTTAGAGCTACCGCA-3′
sgACTB	GenScript	5′-CCGCCTAGAAGCATTTGCGG-3′

shRNA

TARGET	SOURCE	CATALOG NUMBER
shNTC	Horizon Discovery	Cat# VSC6572 Lot# V20012406
shCdk2 #1	Horizon Discovery	Cat# V3SH7670-229933017; clone ID V3IHSHEG_9870667
shCdk2 #2	Horizon Discovery	Cat# V3SH7669-227445411; clone ID V3IHSHEG_7383061
shCdk1#1	Horizon Discovery	Cat# V3SH7669-229925658; clone ID V3IHSHEG_9863308
shCdk1 #2	Horizon Discovery	Cat# V3SH7669-229395843; clone ID V3IHSHEG_9333493

TABLES

Table S1. *CCNE1* amplification trends toward mutually exclusivity with HRD, FA, and DNA repair pathway mutations

Co-occurrence of *CCNE1* amplification and alterations in genes of interest in patients with ovarian cancer included in the TCGA. Odds ratio, p-value, and tendency were calculated using cBioPortal.

**Gene**	**Category**	**Genes in category**	**Log_2 odds ratio**	***P* value**	**Tendency**
CCNE1	HR deficient	*BRCA1, BRCA2, CDK12, RAD50, RAD52, RAD54L*	-1.03	0.1148	Mutual exclusivity
CCNE1	Fanconi anemia	*PALB2, FANCA, FANCC, FANCI, FANCL*	-1.77	0.2397	Mutual exclusivity
CCNE1	DNA repair	*ATM, ATR, CHEK1, CHEK2*	-1.21	0.2647	Mutual exclusivity
CCNE1	RB1	*RB1*	-1.311	0.057	Mutual exclusivity
CCNE1	PIK3CA	*PIK3CA*	0.10	0.4425	Co-occurrence

ATM, ataxia-telangiectasia mutated; ATR, ataxia telangiectasia and Rad3 related; BRCA1, breast cancer type 1; BRCA2, breast cancer type 2; CCNE1, cyclin E1; CDK12, cyclin-dependent kinase 12; CHEK 1, checkpoint kinase 1; CHEK 2, checkpoint kinase 2; FA, Fanconi anemia; FANCA, Fanconi anemia, complementation group A; FANCC, Fanconi anemia, complementation group C; FANCI, Fanconi anemia, complementation group I; FANCL, Fanconi anemia, complementation group L; HR, homologous recombination; HRD, homologous recombination deficiency; PALB2, partner and localizer of BRCA2; PIK3CA, phosphatidylinositol-4,5-bisphosphate 3-kinase, catalytic subunit alpha; RAD50, radiation sensitive 50; RAD52, radiation sensitive 50; RAD54L, radiation sensitive 54-like; RB1, RB transcriptional corepressor 1.

Table S2. *CCNE1*, p53, BRCA status for model cell lines

CCNE1 copy number and *p53*, *BRCA1*, *BRCA2*, and *PIK3CA* mutations sourced from CCLE. *CCNE1* copy numbers and p53 status for A549, COV644, OVCAR-3, COV318, and FUOV1 cells retrieved from CCLE DepMap release 21Q2 were reported as log2(copy number/ploidy+1). Retransformed copy number estimates are reported in the *CCNE1* copy number column assuming diploid status. *CCNE1* copy number for hTERT-RPE1 is assumed to be copy number normal based on its diploid status.

Cell line	***CCNE1* copy number**	**p53 status**	**BRCA1/BRCA2/PIK3CA status**
hTERT-RPE1	2.00	WT	WT
A549	3.10	WT	WT
COV644	2.38	WT	WT
OVCAR-3	12.3	R248Q	WT
COV318	6.70	I195F	WT
FUOV1	23.50	H179D	WT

A549, adenocarcinomic human alveolar basal epithelial cells; BRCA1, breast cancer type 1; BRCA2, breast cancer type 2; CCNE1, cyclin E1; COV318, human ovarian epithelial-serous carcinoma cell line established from a peritoneal ascites; COV644, human ovarian epithelial-mucinous carcinoma cell line established from a solid primary tumor; FUOV1, Fukuoka University-OVarian-1; H179D, substitution - missense, position 179, H→D; hTERT-RPE1, telomerase reverse transcriptase immortalized human retinal epithelial cells; I195F, substitution - missense, position 195, I→F; OVCAR-3, high-grade serous ovarian adenocarcinoma cell line established from a patient refractory to cisplatin; p53, tumor protein 53; PIK3CA, phosphatidylinositol-4,5-bisphosphate 3-kinase, catalytic subunit alpha; R248Q, substitution - missense, position 248, R→Q; WT, wild type.

Table S3. BLU1851 is a selective CDK2 inhibitor

(**A**) IC_{50} (nM) of BLU1851 in the ATP-competition caliper enzyme assay in indicated CDK complexes. (**B**) IC_{50} (nM) of BLU1851 in the NanoBRET assay performed in HEK-293T cells transfected with indicated CDK complexes. (**C**) IC_{50} (nM) of indicated compound in MCF-7 and OVCAR-3 proliferation assays. IC_{50}'s represent the mean of two independent replicates.

A

Compound	CDK2/ CycE1	CDK2/ CycA2	CDK1/ CycB1	CDK4/ CycD1	CDK6/ CycD3	CDK7/CycH1/MNAT1	CDK9/ CycT1
BLU1851	4.1	36.4	736.9	30.8	174.4	4,087.5	>10,000

B

Compound	CDK2/ CycE1	CDK1/ CycB1	CDK4/ CycD1	CDK6/ CycD3	CDK7/ CycH1	CDK9/ CycT1
BLU1851	2.3	337.0	214.7	167.5	2,600.3	9,398.3

C

Compound	MCF-7 IC_{50} (nM)	OVCAR-3 IC_{50} (nM)
Palbociclib	149.8	>25,000
BLU1851	1,357.8	282.8

CDK, cyclin-dependent kinase; CDK1, cyclin-dependent kinase 1; CDK2, cyclin-dependent kinase 2; CDK4, cyclin-dependent kinase 4; CDK6, cyclin-dependent kinase 6; CDK7, cyclin-dependent kinase 7; CDK9, cyclin-dependent kinase 9; Cyc, cyclin; CycA2, cyclin A2; CycB1, cyclin B1; CycD1, cyclin D1; CycD3, cyclin D3; CycE1, cyclin E1; CycH1, cyclin H1; CycT1, cyclin T1; IC_{50}, half-maximal drug inhibitory concentration; MNAT1, MNAT CDK-activating kinase assembly factor 1

Table S4. BLU2256 is a selective CDK2 inhibitor

(**A**) IC_{50} (nM) of indicated compound in the ATP-competition caliper enzyme assay. (**B**) IC_{50} (nM) of indicated compound in the NanoBRET assay performed in HEK-293T cells. (**C**) IC_{50} (nM) of indicated compound in MCF-7 and OVCAR-3 proliferation assays. IC_{50}'s represent the mean of two independent replicates.

A

Compound	CDK2/ CycE1	CDK2/ CycA2	CDK1/ CycB1	CDK4/ CycD1	CDK6/ CycD3	CDK7/Cyc H1/MNAT1	CDK9/ CycT1
BLU2256	0.7	11.3	153.6	117.0	394.6	>6,666.7	9,107.0

B

Compound	CDK2/ CycE1	CDK1/ CycB1	CDK4/ CycD1	CDK6/ CycD3	CDK7/ CycH1	CDK9/ CycT1
BLU2256	5.3	84.9	579.1	735.5	4,422.9	3,702.6

C

Compound	MCF-7 IC_{50} (nM)	OVCAR-3 IC_{50} (nM)
Palbociclib	149.8	>25,000
BLU2256	1,282.4	82.5

CDK, cyclin-dependent kinase; CDK1, cyclin-dependent kinase 1; CDK2, cyclin-dependent kinase 2; CDK4, cyclin-dependent kinase 4; CDK6, cyclin-dependent kinase 6; CDK7, cyclin-dependent kinase 7; CDK9, cyclin-dependent kinase 9; Cyc, cyclin; CycA2, cyclin A2; CycB1, cyclin B1; CycD1, cyclin D1; CycD3, cyclin D3; CycE1, cyclin E1; CycH1, cyclin H1; CycT1, cyclin T1; IC_{50}, half-maximal drug inhibitory concentration; MNAT1, MNAT CDK-activating kinase assembly factor 1.

SUPPLEMENTARY FIGURES

Figure S1. *CCNE1*-amplified ovarian cancer cell lines are prone to GCR

(**A**) Percent of cell lines with at least one fusion event within a known fragile site in amplified and non-amplified cancer cell lines (n=1371) (Fisher's Exact Test, *P* = 0.03). (**B**) Percent of cell lines with at least one fusion event within a known fragile site in amplified and non-amplified ovarian cancer cell lines (n=64) (Fisher's Exact Test, *P* = 0.16). (**C**) Difference in estimated tumor mutation burden in *CCNE1*-amplified and non-amplified cancer cell lines (n=1371) (Wilcoxon Rank Sum Test, *P* = 0.74). (**D**) Difference in estimated tumor mutation burden in *CCNE1*-amplified and non-amplified ovarian cancer cell lines (n=64) (Wilcoxon Rank Sum Test, *P* = 0.79).

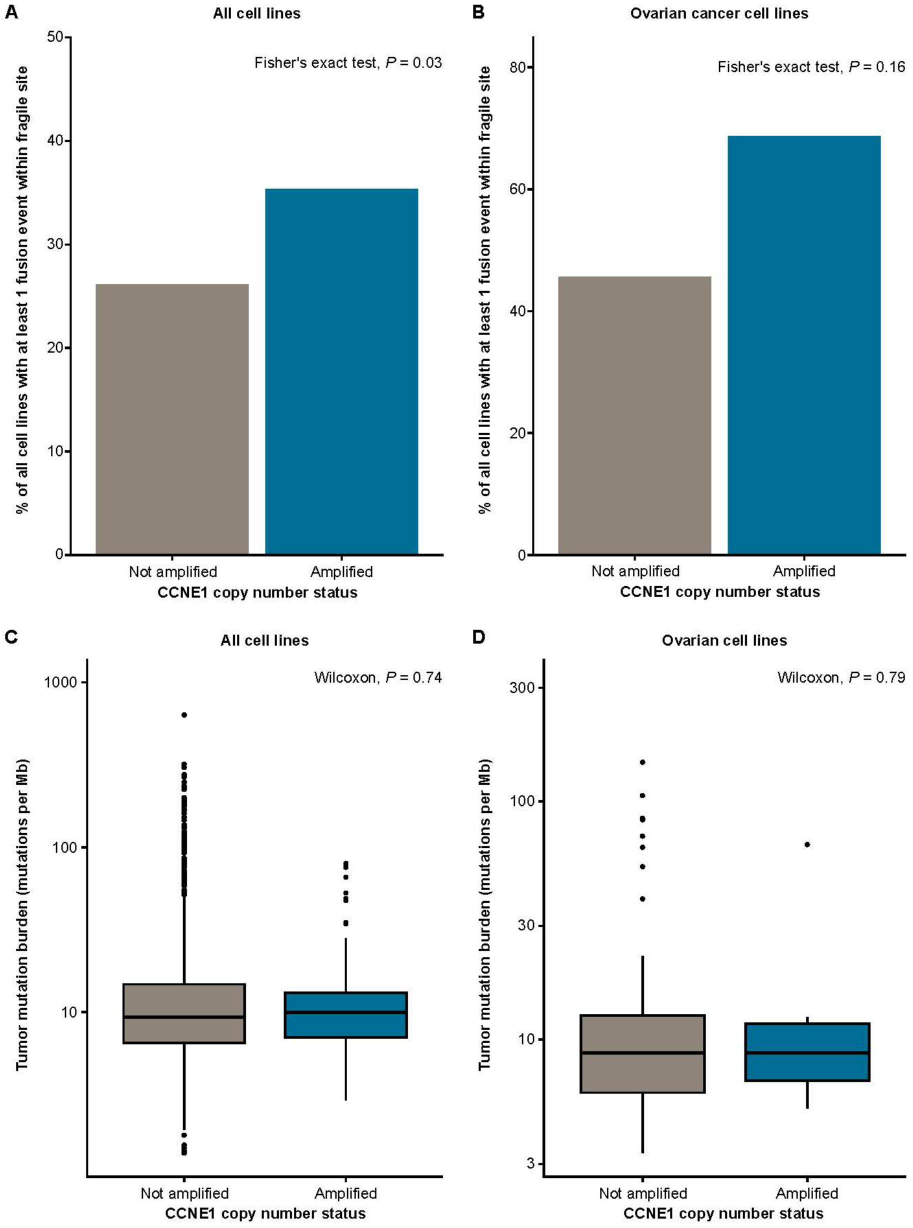

Figure S2. Replication fork stall signaling is dependent on CDK2, HR in *CCNE1*-amplified cells

(A) Cyclin E1 protein expression is strongly associated with *CCNE1* copy number amplification among TCGA patients with ovarian cancer (n=304) (Welch Two Sample t-test, P = 2.3E-13). (**B**) Chemical structures of BLU1851. (**C**) Kinome tree for BLU1851 at 3 μM (S-Score = 0.050). (**D**) Dose-response curves for BLU1851 enzyme inhibition for indicated CDK family members. Error bars represent SD of at least duplicate independent experiments. (**E**) NanoBRET assessment of BLU1851 target engagement in indicated CDK complexes expressed in HEK-293 cells. Error bars represent SD of at least duplicate independent experiments. (**F**) Dose-response curve for pRb T821/826 inhibition by BLU1851 in OVCAR-3 cells. Error bars represent SD of at least duplicate independent experiments. (**G**) Dose-response curve for pLamin S22 inhibition by BLU1851 in OVCAR-3 and COV644 cells. Error bars represent SD of duplicate independent experiments. (**H**) Dose-response curves in MCF-7, OVCAR-3 cells. Cells were treated with a 10-point dose response of indicated compounds and incubated for 5 days before CyQuant assay was performed. Error bars represent SD of at least duplicate independent experiments. (**I**) Quantification of pCHK1 S317 normalized to actin and to DMSO treated vehicle samples from Figure 2C. Error bars represent SD in three independent experiments. Statistical analysis: Student's T-test, * = P < 0.05, ** = P < 0.01, *** = P < 0.001, **** = P < 0.0001, ns = not significant. (**J**) Western blot analysis of indicated antibodies in FUOV1, COV318, hTERT-RPE1, and A549 cells. Cells were treated with BLU1851 (100 nM) for 24 hours prior to addition of hydroxyurea (HU) (2 mM) at indicated time points. (**K**) Confluency of OVCAR-3 at experimental endpoint. OVCAR-3 cells were treated for 2 hours with rabusertib (500 nM) before the addition of HU (2 mM) for 24 hours. After 24 hours, HU was washed out and cells were allowed to recover in the presence of rabusertib. Confluence was calculated using IncuCyte. Error bars represent SD in duplicate experiments. Statistical analysis: Student's t-test, *** = P < 0.001, **** = P < 0.0001, ns = not significant. (**L**) Western blot analysis of indicated antibodies. OVCAR-3 cells were treated as in Figure 4E in an independent experiment, and collected at day 7 after initial transfection. (**M**) Western blot analysis of indicated antibodies. COV644 cells were transfected with non-targeting siRNA (NTC), RAD51 siRNA, or REV1 siRNA, and collected 48 hours later. (**N**) Cell confluency at experimental endpoint. Confluence was measured by IncuCyte live cell imaging and values were determined using an algorithm to detect cells from phase-contrast images. Cells were transfected with siRNA 24 hours before addition of HU (2 mM) for 24 hours. After the HU was washed out, cells were transfected again with indicated siRNAs and allowed to incubate for 5 additional days. HU-treated groups were normalized to vehicle-treated groups for each siRNA at the endpoint. The dotted line represents confluency of cells treated with constant HU exposure over the time course of the experiment. Error bars represent SD in duplicate experiments. Statistical analysis: Student's t-test, * = P < 0.05, ** = P < 0.01, *** = P < 0.001, **** = P < 0.0001, ns = not significant.

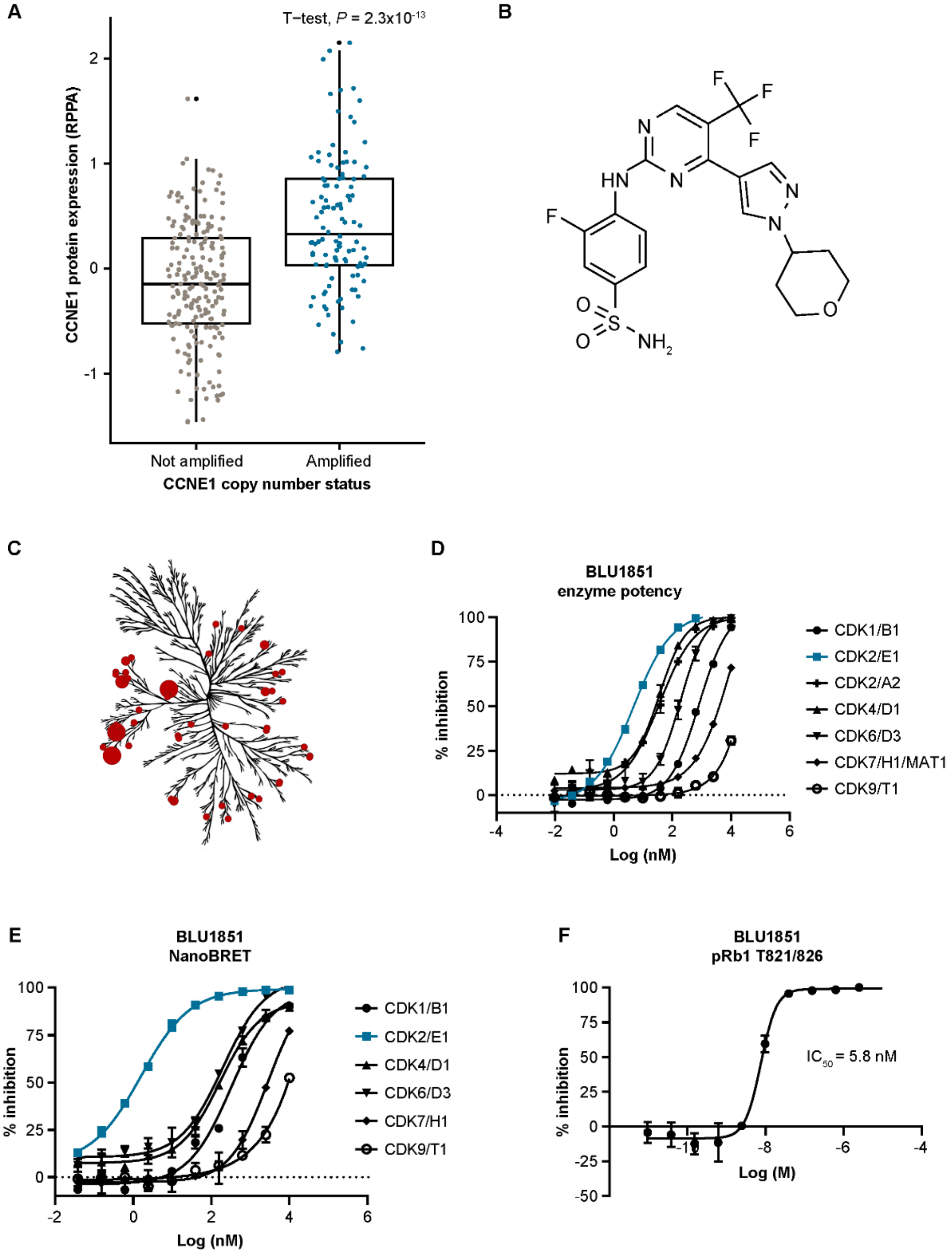
A
T−test, P = 2.3x10-13
CCNE1 protein expression (RPPA)
Not amplified
Amplified
CCNE1 copy number status
B
C
D
BLU1851
enzyme potency
% inhibition
Log (nM)
CDK1/B1
CDK2/E1
CDK2/A2
CDK4/D1
CDK6/D3
CDK7/H1/MAT1
CDK9/T1
E
BLU1851
NanoBRET
% inhibition
Log (nM)
CDK1/B1
CDK2/E1
CDK4/D1
CDK6/D3
CDK7/H1
CDK9/T1
F
BLU1851
pRb1 T821/826
% inhibition
Log (M)
IC50 = 5.8 nM

G

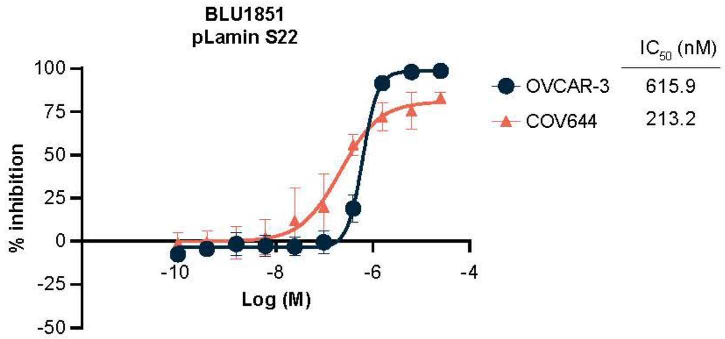
BLU1851
pLamin S22
% inhibition
Log (M)
OVCAR-3
COV644
IC50 (nM)
615.9
213.2

H

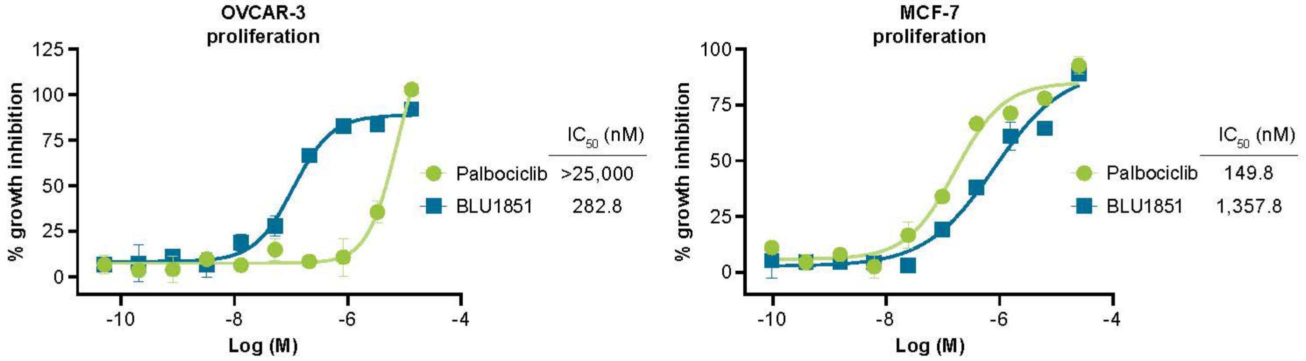
OVCAR-3
proliferation
% growth inhibition
Log (M)
Palbociclib
BLU1851
IC50 (nM)
>25,000
282.8
MCF-7
proliferation
% growth inhibition
Log (M)
Palbociclib
BLU1851
IC50 (nM)
149.8
1,357.8

I

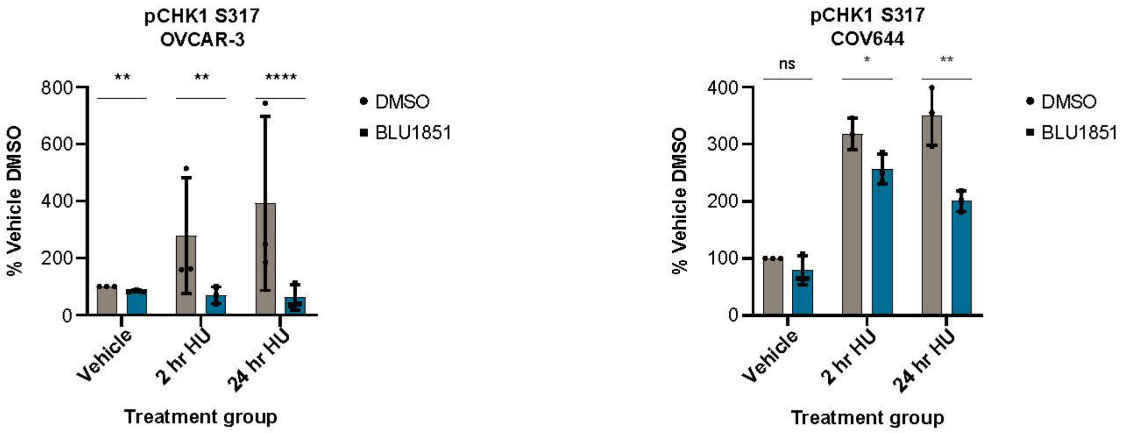
pCHK1 S317
OVCAR-3
% Vehicle DMSO
Vehicle
2 hr HU
24 hr HU
Treatment group
DMSO
BLU1851
pCHK1 S317
COV644
% Vehicle DMSO
ns
Vehicle
2 hr HU
24 hr HU
Treatment group
DMSO
BLU1851

J

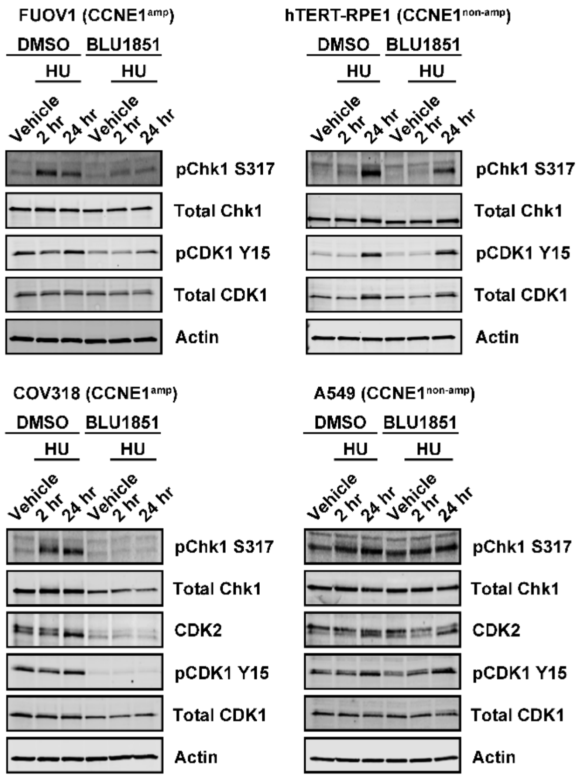
FUOV1 (CCNE1amp)
DMSO
BLU1851
HU
HU
Vehicle
2 hr
24 hr
Vehicle
2 hr
24 hr
pChk1 S317
Total Chk1
pCDK1 Y15
Total CDK1
Actin
hTERT-RPE1 (CCNE1non-amp)
DMSO
BLU1851
HU
HU
Vehicle
2 hr
24 hr
Vehicle
2 hr
24 hr
pChk1 S317
Total Chk1
pCDK1 Y15
Total CDK1
Actin
COV318 (CCNE1amp)
DMSO
BLU1851
HU
HU
Vehicle
2 hr
24 hr
Vehicle
2 hr
24 hr
pChk1 S317
Total Chk1
CDK2
pCDK1 Y15
Total CDK1
Actin
A549 (CCNE1non-amp)
DMSO
BLU1851
HU
HU
Vehicle
2 hr
24 hr
Vehicle
2 hr
24 hr
pChk1 S317
Total Chk1
CDK2
pCDK1 Y15
Total CDK1
Actin

K

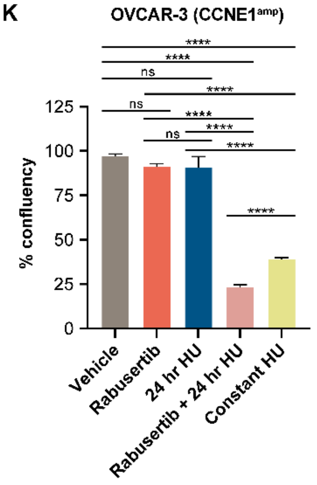
OVCAR-3 (CCNE1amp)
****
****
ns
****
ns
****
ns
****
****
****
125
100
75
50
25
0
% confluency
Vehicle
Rabusertib
24 hr HU
Rabusertib + 24 hr HU
Constant HU

L

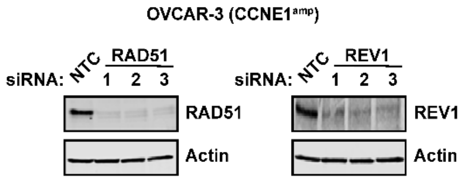
OVCAR-3 (CCNE1amp)
siRNA: NTC
RAD51
1 2 3
RAD51
Actin
siRNA: NTC
REV1
1 2 3
REV1
Actin

M

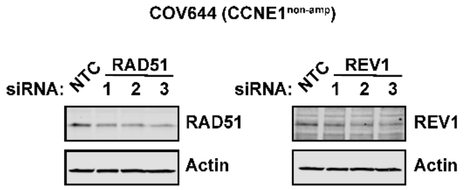
COV644 (CCNE1non-amp)
siRNA: NTC
RAD51
1 2 3
RAD51
Actin
siRNA: NTC
REV1
1 2 3
REV1
Actin

N

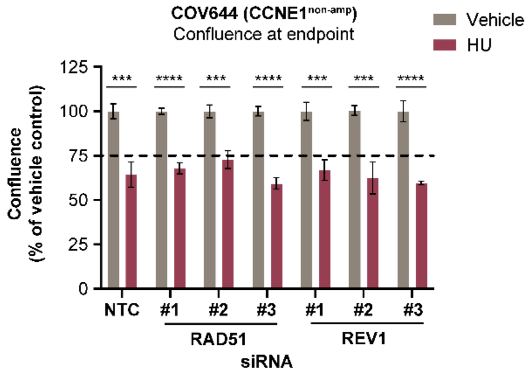
COV644 (CCNE1non-amp)
Confluence at endpoint
Vehicle
HU
***
****
***
****
***
***
****
125
100
75
50
25
0
Confluence
(% of vehicle control)
NTC
#1
#2
#3
#1
#2
#3
RAD51
REV1
siRNA

Figure S3. CDK2 and cyclin E1 is recruited to stalled replication forks in *CCNE1*-amplified cells

(**A**) Representative images of PLA for each noted interaction in FUOV1 and hTERT-RPE1 cells. (**B**) The negative control excluded 1 primary antibody. The graph shows the mean number of PLA spots per cell in each PLA assay. Bars represent the mean (ns = not significant, * = *P* < 0.05, ** = *P* < 0.01, *** = *P* < 0.001, **** *P* < 0.0001, Mann-Whitney test; n = at least 75 cells/experiment).

A

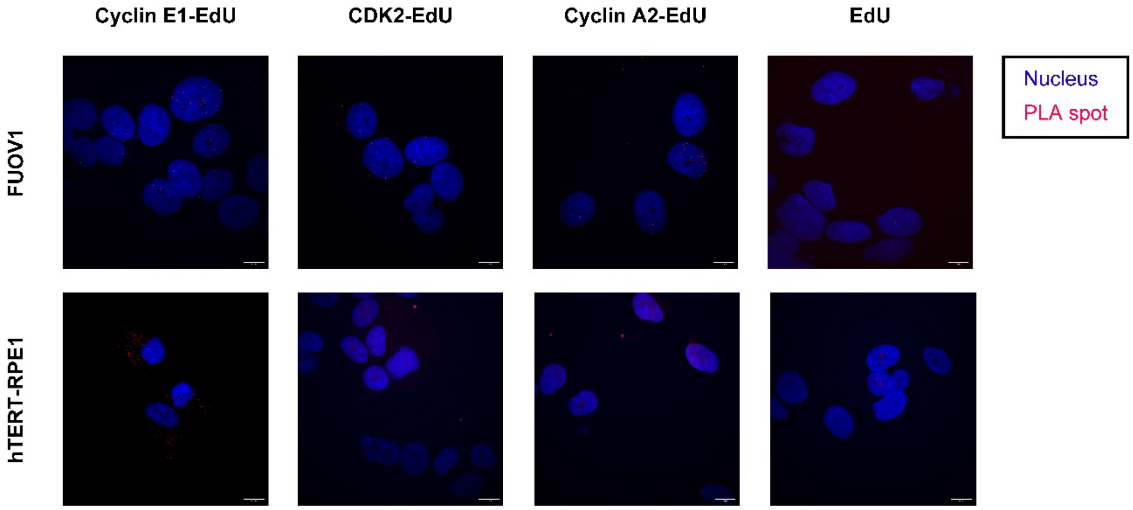

B

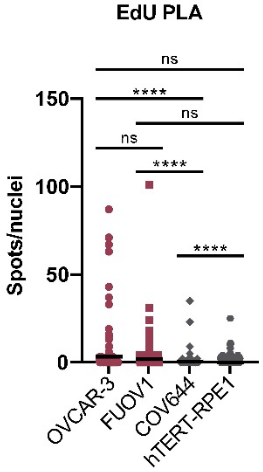

Figure S4. CDK2 regulates HR in *CCNE1*-amplified cell lines

(**A**) Quantification of pRPA normalized to actin and to Etoposide treated shNTC from Figure 4A. Error bars represent SD in three independent experiments. Statistical analysis: Student's T-test, * = P < 0.05, ** = P < 0.01, *** = P < 0.001, **** = P < 0.0001, ns = not significant. (**B**) Western blot analysis with the indicated antibodies. Small hairpin RNAs (shRNAs) targeting CDK1 were induced by doxycycline (dox) for 96 hours prior to etoposide (E) treatment at 50 µM for 2 hours in OVCAR-3 or COV644 cells. (**C**) Western blot analysis with the indicated antibodies. shRNAs targeting CDK2, CDK1, or a non-targeting control (NTC) were induced by doxycycline (dox) for 96 hours prior to etoposide (E) treatment at 50 µM for 2 hours in FUOV1 cells. (**D**) Western blot analysis with the indicated antibodies. shRNAs targeting CDK2, CDK1, or a NTC were induced by dox for 96 hours prior to etoposide (E) treatment at 50 µM for 2 hours in COV318 cells. (**E**) Western blot analysis with the indicated antibodies. shRNAs targeting CDK2, CDK1, or a NTC were induced by doxycycline (dox) for 96 hours prior to etoposide (E) treatment at 50 µM for 2 hours in A549 cells. (**F**) Western blot analysis with the indicated antibodies. shRNAs targeting CDK2, CDK1, or a NTC were induced by doxycycline (dox) for 96 hours prior to etoposide (E) treatment at 50 µM for 2 hours in hTERT-RPE1 cells. (**G**) Quantification of pRPA normalized to actin from Figure 4B. Error bars represent SD in three independent experiments. Statistical analysis: Student's T-test, * = $P < 0.05$, ** = $P < 0.01$, *** = $P < 0.001$, **** = $P < 0.0001$, ns = not significant. (**H**) Western blot analysis with the indicated antibodies. Cells were treated with indicated 25 nM or 25 0nM BLU1851, 1 µM RO-3306, or 10 µM roscovitine for 24 hours before being treated with 50 µM etoposide (E) for 2 hours. (**I**) Western blot analysis with indicated antibodies. Cells were treated with 25 nM or 250 nM BLU1851, 1 µM RO-3306, or 10 µM roscovitine. Contemporaneously, cells were transfected with a 3X-FLAG donor plasmid plus a plasmid containing Cas9 and a single guide RNA (sgRNA) scramble control (sgScr) or a sgRNA targeting beta-actin (sgACTB). Cells were harvested 72 hours later. (**J**) Cell cycle profiles after 24 hours of treatment with BLU1851 at indicated concentrations in asynchronously growing cells. Error bars represent SD in duplicate independent experiments. (**K**) Cell cycle profile of OVCAR-3 cells after double-thymidine block or 24 hour treatment with 250 nM BLU1851. Error bars represent SEM of duplicate independent experiments. (**L**) Western blot analysis with indicated antibodies after double-thymidine block or 24-hour treatment with 250 nM BLU1851.

A

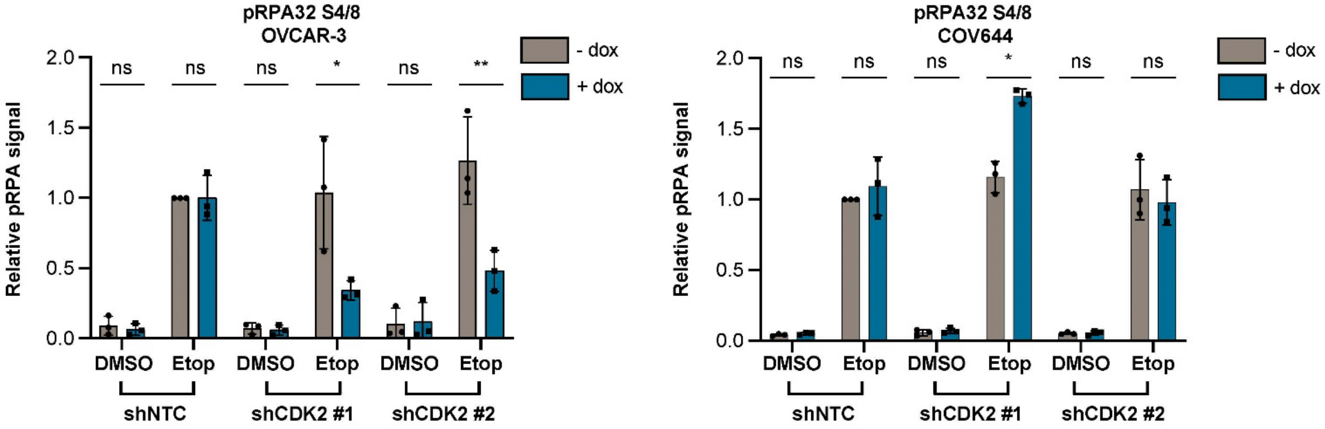
pRPA32 S4/8
OVCAR-3
Relative pRPA signal
2.0
1.5
1.0
0.5
0.0
ns
ns
ns
*
ns
**
- dox
+ dox
DMSO
Etop
DMSO
Etop
DMSO
Etop
shNTC
shCDK2 #1
shCDK2 #2
pRPA32 S4/8
COV644
Relative pRPA signal
2.0
1.5
1.0
0.5
0.0
ns
ns
ns
*
ns
ns
- dox
+ dox
DMSO
Etop
DMSO
Etop
DMSO
Etop
shNTC
shCDK2 #1
shCDK2 #2

B

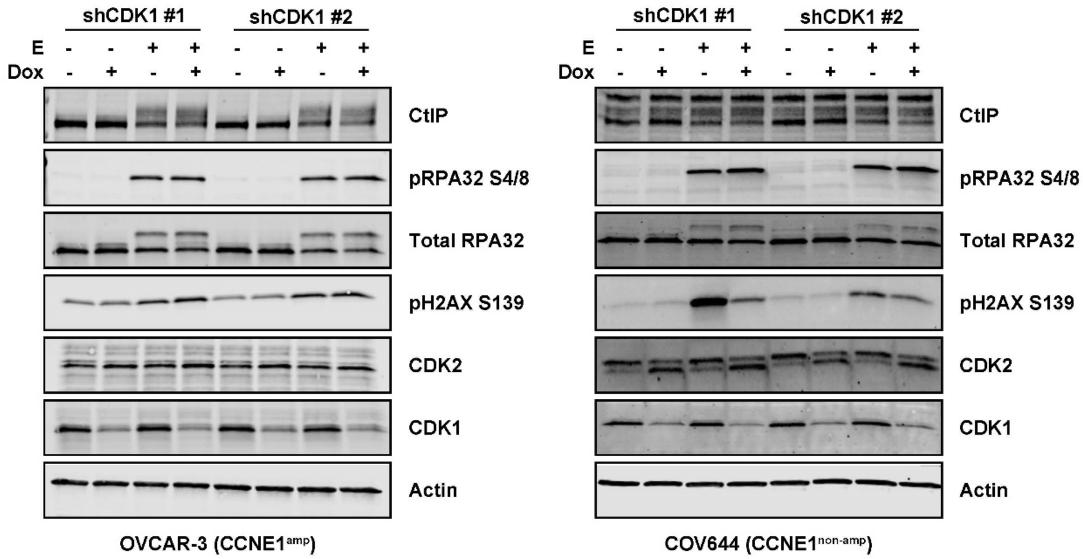
shCDK1 #1
shCDK1 #2
E - - + + - - + +
Dox - + - + - + - +
CtIP
pRPA32 S4/8
Total RPA32
pH2AX S139
CDK2
CDK1
Actin
OVCAR-3 (CCNE1amp)
shCDK1 #1
shCDK1 #2
E - - + + - - + +
Dox - + - + - + - +
CtIP
pRPA32 S4/8
Total RPA32
pH2AX S139
CDK2
CDK1
Actin
COV644 (CCNE1non-amp)

C

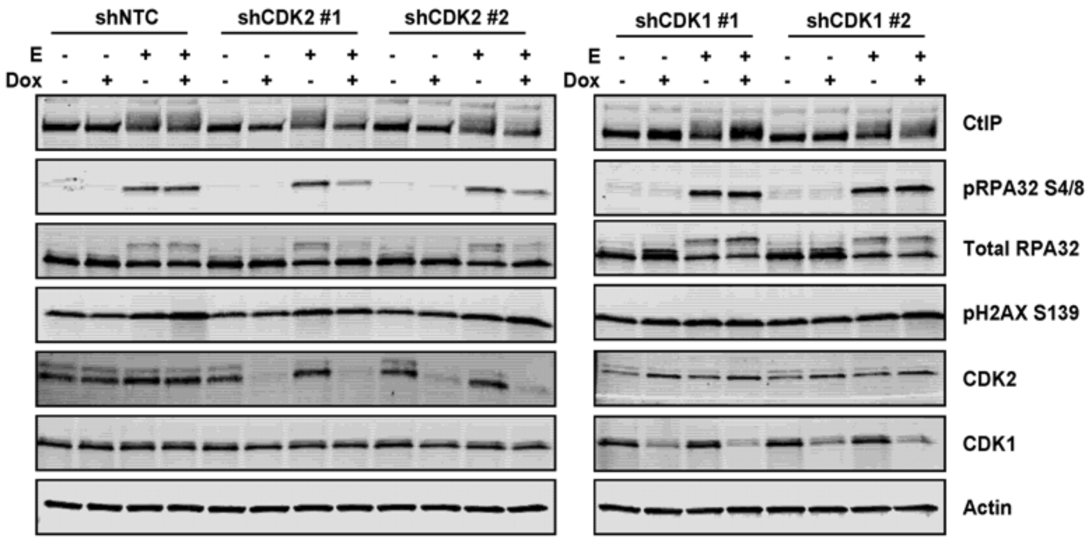

FUOV1 (CCNE1[amp])

D

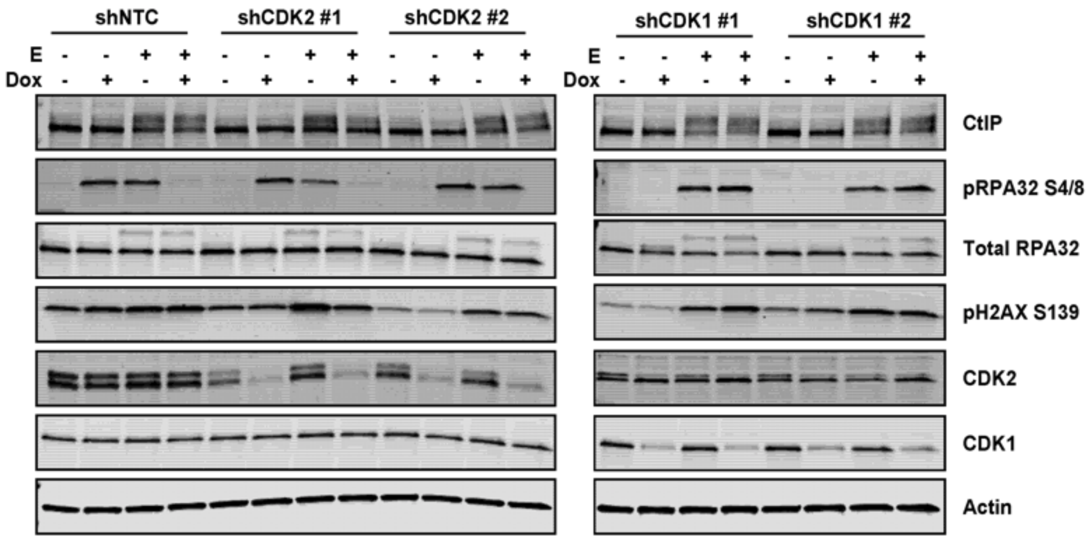

COV318 (CCNE1[amp])

E

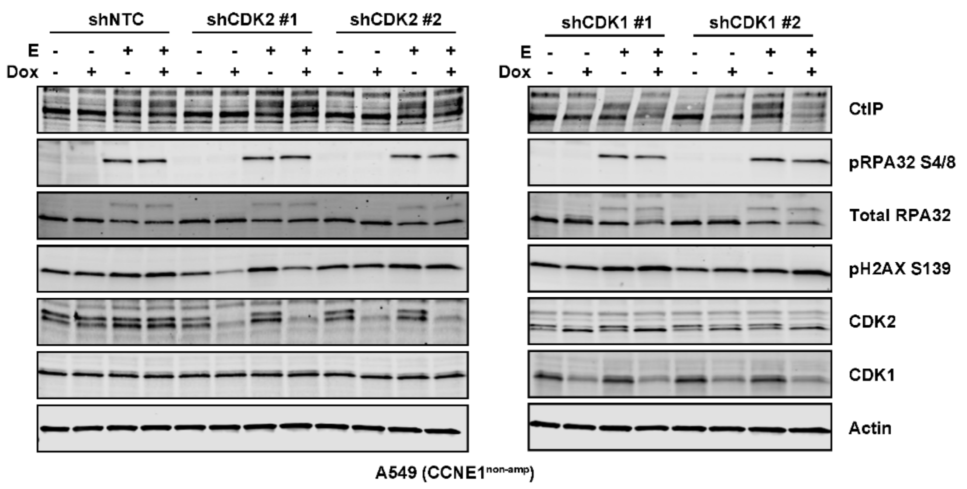
shNTC
shCDK2 #1
shCDK2 #2
shCDK1 #1
shCDK1 #2
E
Dox
CtIP
pRPA32 S4/8
Total RPA32
pH2AX S139
CDK2
CDK1
Actin
A549 (CCNE1non-amp)

F

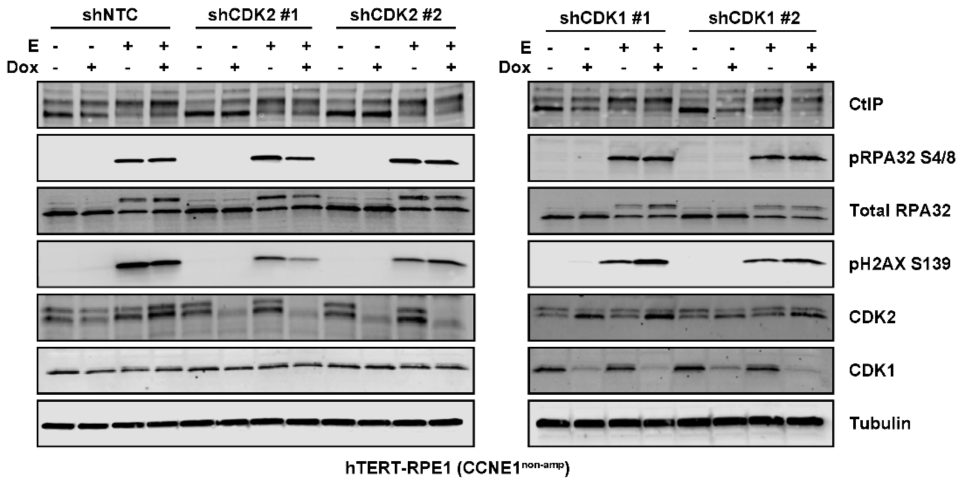
shNTC
shCDK2 #1
shCDK2 #2
shCDK1 #1
shCDK1 #2
E
Dox
CtIP
pRPA32 S4/8
Total RPA32
pH2AX S139
CDK2
CDK1
Tubulin
hTERT-RPE1 (CCNE1non-amp)

G

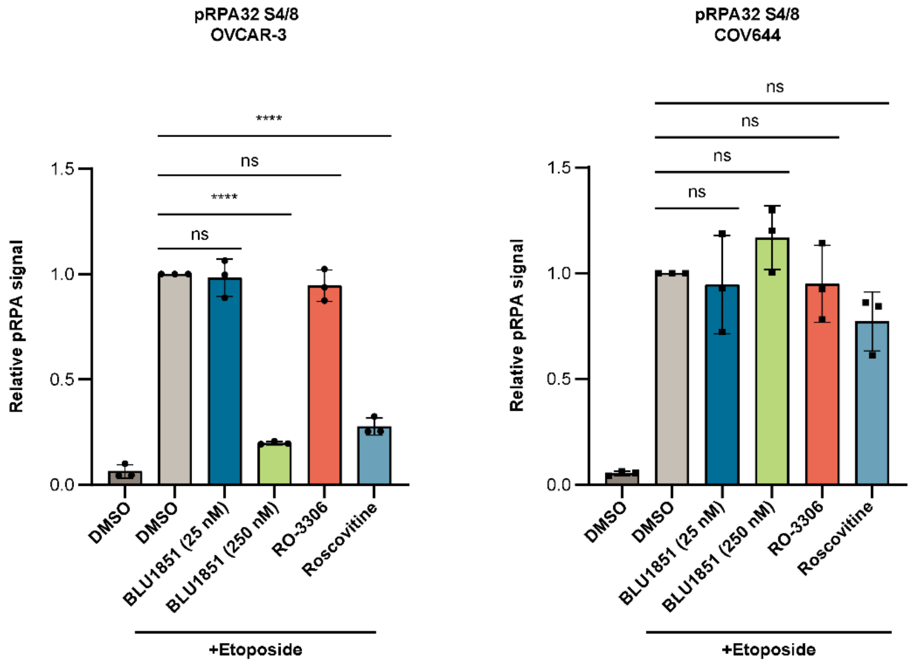
pRPA32 S4/8
OVCAR-3
pRPA32 S4/8
COV644
Relative pRPA signal
0.0
0.5
1.0
1.5
ns
****
DMSO
DMSO
BLU1851 (25 nM)
BLU1851 (250 nM)
RO-3306
Roscovitine
+Etoposide

H

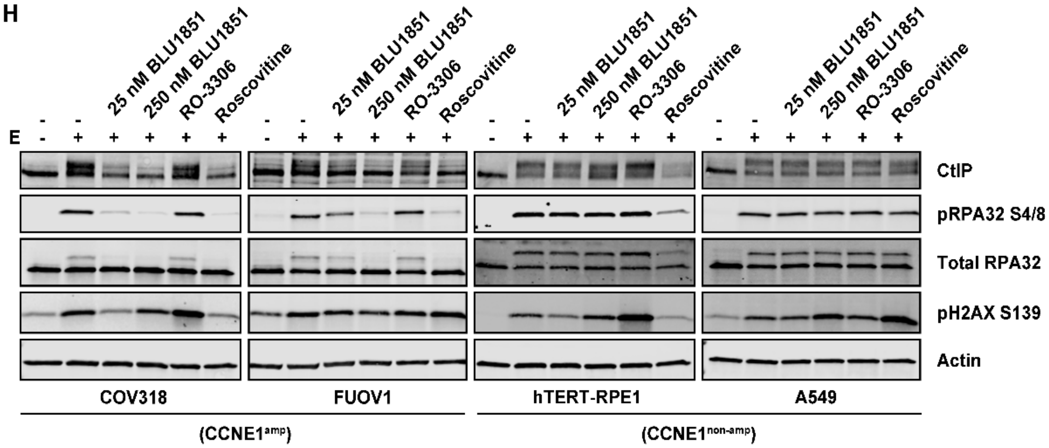
25 nM BLU1851
250 nM BLU1851
RO-3306
Roscovitine
E
CtIP
pRPA32 S4/8
Total RPA32
pH2AX S139
Actin
COV318
FUOV1
hTERT-RPE1
A549
(CCNE1amp)
(CCNE1non-amp)

I

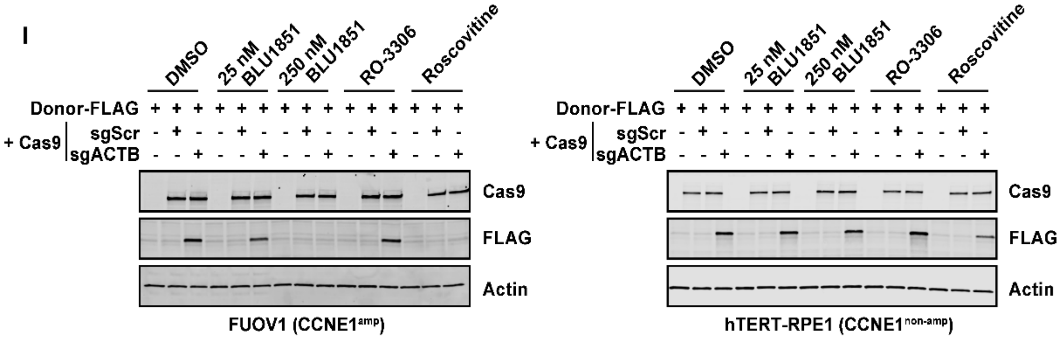
DMSO
25 nM BLU1851
250 nM BLU1851
RO-3306
Roscovitine
Donor-FLAG
+ Cas9
sgScr
sgACTB
Cas9
FLAG
Actin
FUOV1 (CCNE1amp)
hTERT-RPE1 (CCNE1non-amp)

J

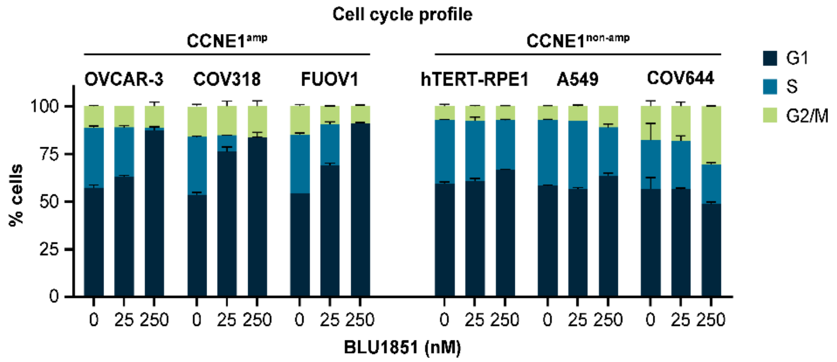
Cell cycle profile
CCNE1amp
CCNE1non-amp
OVCAR-3
COV318
FUOV1
hTERT-RPE1
A549
COV644
G1
S
G2/M
% cells
100
75
50
25
0
0 25 250
BLU1851 (nM)

K

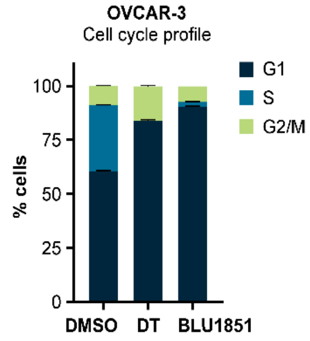
OVCAR-3
Cell cycle profile
G1
S
G2/M
% cells
100
75
50
25
0
DMSO
DT
BLU1851

L

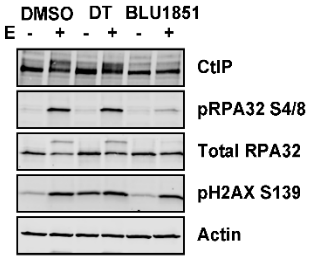
DMSO
DT
BLU1851
E
CtIP
pRPA32 S4/8
Total RPA32
pH2AX S139
Actin

Figure S5. BLU2256 is a selective CDK2 inhibitor

(**A**) Chemical structures of BLU2256. (**B**) Kinome tree for BLU2256 at 3 µM (S-Score = 0.040). (**C**) Dose-response curves for BLU2256 enzyme inhibition for indicated CDK family members. Error bars represent SD of at least duplicate independent experiments. (**D**) NanoBret assessment of BLU2256 target engagement in indicated CDK complexes expressed in HEK-293 cells. Error bars represent SD of at least duplicate independent experiments. (**E**) Dose-response curve for pRb T821/826 inhibition by BLU2256 in OVCAR-3 cells. Error bars represent SD of at least duplicate independent experiments. (**F**) Dose-response curve for pLamin S22 inhibition by BLU1851 in OVCAR-3 and COV644 cells. Error bars represent SD of duplicate independent experiments. (**G**) Dose-response curves in MCF-7, OVCAR-3 cells. Cells were treated with a 10-point dose response of indicated compounds and incubated for 5 days before CyQuant assay was performed. Error bars represent SD of at least duplicate independent experiments. (**H**) Plasma concentrations of BLU2256 or BLU1851 at indicated doses. NOD-SCID mice were dosed once p.o. with indicated compound and plasma was collected at 0.5, 2, 4, 8, and 24 hours. The dashed lines indicate the *in vitro* proliferative IC_{50} in OVCAR-3 cells corrected for plasma protein binding. Error bars represent SEM. N = 3 mice/timepoint. (**I**) Western blot analysis with the indicated antibodies. OVCAR-3 cells were treated with indicated concentrations of BLU2256 for 24 hours before being treated with 50 µM etoposide (E) for 2 hours. (**J**) Western blot analysis with indicated antibodies. OVCAR-3 cells were treated with 25 nM or 250 nM BLU2256. Contemporaneously, cells were transfected with a 3X-FLAG donor plasmid plus a plasmid containing Cas9 and a single guide RNA (sgRNA), scramble control (sgScr), or an sgRNA targeting beta-actin (sgACTB). Cells were harvested 72 hours later. (**K**) Relative body weight over treatment time. The percent body weight change is calculated by normalizing to day 0 per treatment group. Error bars represent SEM. N = 8 mice/group. (**L**) Western blot analysis of indicated antibodies from tumors treated with BLU2256 at indicated doses. (**M**) Quantification of Western blot for pRb S807/811 normalized individually to actin and then to vehicle group to calculate % inhibition. Error bars represent SEM. N = at least 3/group. BW, body weight. **(N)** Relative body weight over treatment time. The percent body weight change is calculated by normalizing to day 0 per treatment group. Error bars represent SEM. N = 8 mice/group. BW, body weight. (**O**) Relative body weight over treatment time. The percent body weight change is calculated by normalizing to day 0 per treatment group. Error bars represent SEM. N = 8-10 mice/group. BW, body weight.

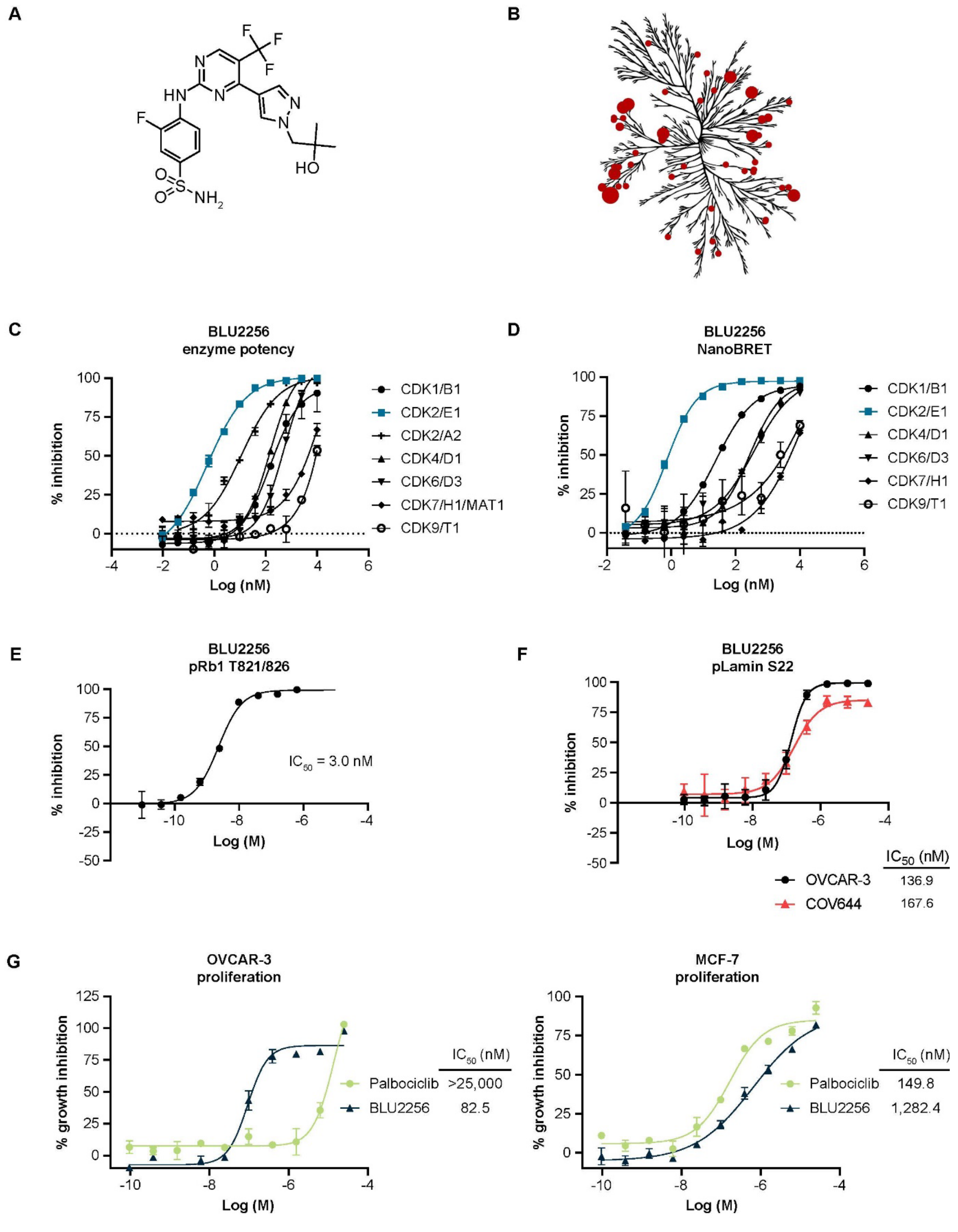
A
B
C
BLU2256
enzyme potency
% inhibition
Log (nM)
CDK1/B1
CDK2/E1
CDK2/A2
CDK4/D1
CDK6/D3
CDK7/H1/MAT1
CDK9/T1
D
BLU2256
NanoBRET
% inhibition
Log (nM)
CDK1/B1
CDK2/E1
CDK4/D1
CDK6/D3
CDK7/H1
CDK9/T1
E
BLU2256
pRb1 T821/826
% inhibition
Log (M)
IC50 = 3.0 nM
F
BLU2256
pLamin S22
% inhibition
Log (M)
IC50 (nM)
OVCAR-3 136.9
COV644 167.6
G
OVCAR-3
proliferation
% growth inhibition
Log (M)
IC50 (nM)
Palbociclib >25,000
BLU2256 82.5
MCF-7
proliferation
% growth inhibition
Log (M)
IC50 (nM)
Palbociclib 149.8
BLU2256 1,282.4

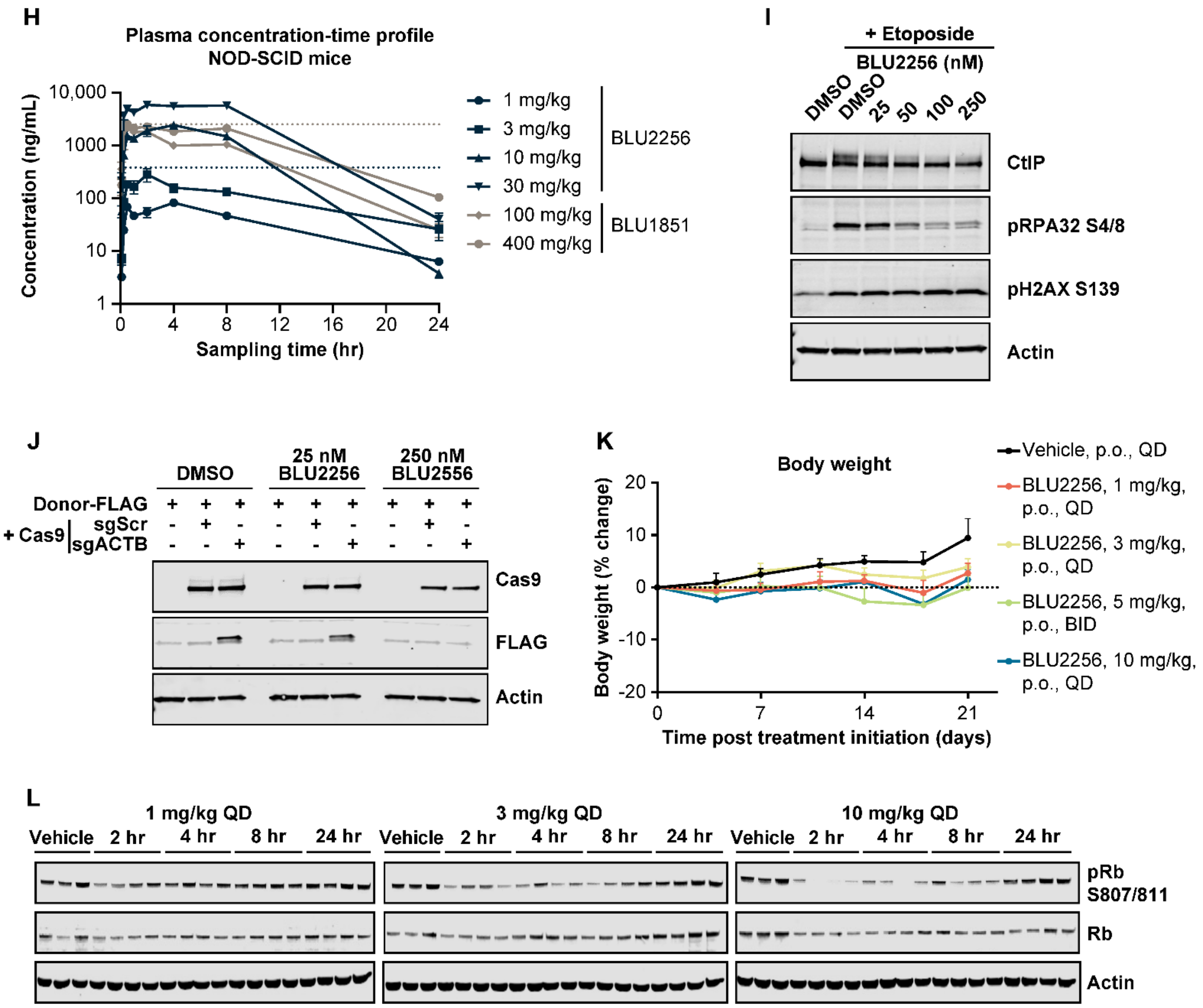
H
Plasma concentration-time profile
NOD-SCID mice
Concentration (ng/mL)
10,000
1000
100
10
1
0
4
8
12
16
20
24
Sampling time (hr)
1 mg/kg
3 mg/kg
10 mg/kg
30 mg/kg
BLU2256
100 mg/kg
400 mg/kg
BLU1851
I
+ Etoposide
BLU2256 (nM)
DMSO
DMSO
25
50
100
250
CtIP
pRPA32 S4/8
pH2AX S139
Actin
J
DMSO
25 nM BLU2256
250 nM BLU2556
Donor-FLAG
+ Cas9
sgScr
sgACTB
Cas9
FLAG
Actin
K
Body weight
Body weight (% change)
20
10
0
-10
-20
0
7
14
21
Time post treatment initiation (days)
Vehicle, p.o., QD
BLU2256, 1 mg/kg, p.o., QD
BLU2256, 3 mg/kg, p.o., QD
BLU2256, 5 mg/kg, p.o., BID
BLU2256, 10 mg/kg, p.o., QD
L
1 mg/kg QD
3 mg/kg QD
10 mg/kg QD
Vehicle
2 hr
4 hr
8 hr
24 hr
pRb S807/811
Rb
Actin

M

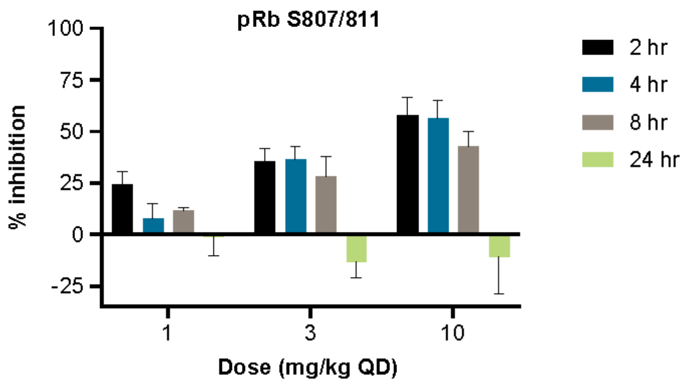
pRb S807/811
% inhibition
100
75
50
25
0
-25
1
3
10
Dose (mg/kg QD)
2 hr
4 hr
8 hr
24 hr

N

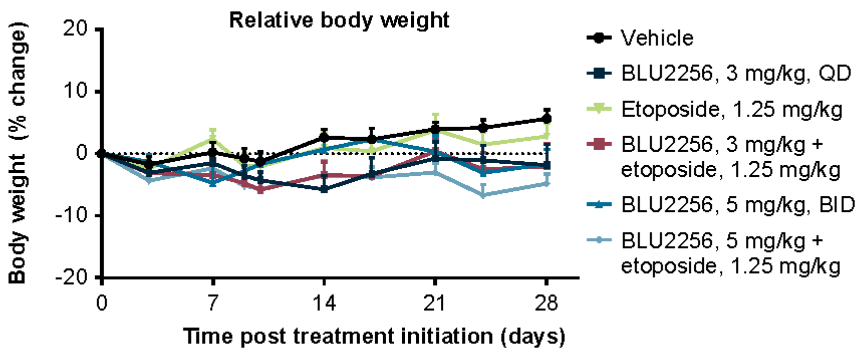
Relative body weight
Body weight (% change)
20
10
0
-10
-20
0
7
14
21
28
Time post treatment initiation (days)
Vehicle
BLU2256, 3 mg/kg, QD
Etoposide, 1.25 mg/kg
BLU2256, 3 mg/kg + etoposide, 1.25 mg/kg
BLU2256, 5 mg/kg, BID
BLU2256, 5 mg/kg + etoposide, 1.25 mg/kg

O

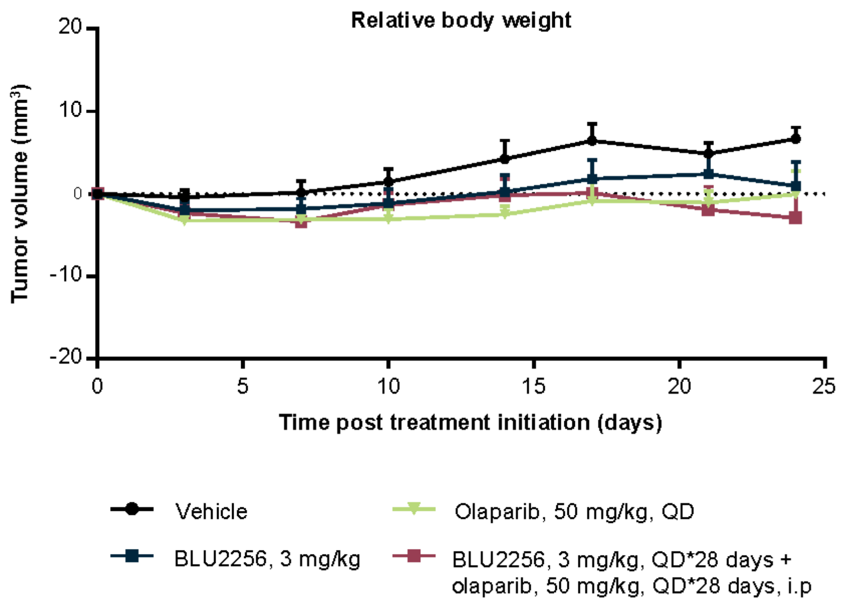
Relative body weight
Tumor volume (mm³)
20
10
0
-10
-20
0
5
10
15
20
25
Time post treatment initiation (days)
Vehicle
Olaparib, 50 mg/kg, QD
BLU2256, 3 mg/kg
BLU2256, 3 mg/kg, QD*28 days + olaparib, 50 mg/kg, QD*28 days, i.p
